# Analysis of m7G-Related signatures in the tumour immune microenvironment and identification of clinical prognostic regulators in breast cancer

**DOI:** 10.1186/s12885-023-11012-z

**Published:** 2023-06-23

**Authors:** Qinghua Huang, Jianlan Mo, Huawei Yang, Yinan Ji, Rong Huang, Yan Liu, You Pan

**Affiliations:** 1 Department of Breast Surgery, Key Laboratory of Breast Cancer Diagnosis and Treatment Research of Guangxi Department of Education, Guangxi Medical University Cancer Hospital, Nanning, 530000 China; 2 Key Laboratory of Breast Cancer Diagnosis and Treatment Research of Guangxi Department of Education, Guangxi Medical University Cancer Hospital, Nanning, 530000 P.R. China; 3 Department of Anesthesiology, Maternal and Child Health Hospital of Guangxi Zhuang Autonomous Region, Nanning, China; 4 Department of BreastBone and Soft Tissue Oncology, Guangxi Medical University Cancer Hospital, Nanning, 530000 China

**Keywords:** N7-methylguanosine, Breast cancer, Immune microenvironment, Prognostic signature, qRT-PCR

## Abstract

**Background:**

Breast cancer is a malignant tumour that seriously threatens women’s life and health and exhibits high inter-individual heterogeneity, emphasising the need for more in-depth research on its pathogenesis. While internal 7-methylguanosine (m7G) modifications affect RNA processing and function and are believed to be involved in human diseases, little is currently known about the role of m7G modification in breast cancer.

**Methods and Results:**

We elucidated the expression, copy number variation incidence and prognostic value of 24 m7G-related genes (m7GRGs) in breast cancer. Subsequently, based on the expression of these 24 m7GRGs, consensus clustering was used to divide tumour samples from the TCGA-BRCA dataset into four subtypes based on significant differences in their immune cell infiltration and stromal scores. Differentially expressed genes between subtypes were mainly enriched in immune-related pathways such as ‘Ribosome’, ‘TNF signalling pathway’ and ‘*Salmonella* infection’. Support vector machines and multivariate Cox regression analysis were applied based on these 24 m7GRGs, and four m7GRGs—AGO2, EIF4E3, DPCS and EIF4E—were identified for constructing the prediction model. An ROC curve indicated that a nomogram model based on the risk model and clinical factors had strong ability to predict the prognosis of breast cancer. The prognoses of patients in the high- and low-TMB groups were significantly different (*p* = 0.03). Moreover, the four-gene signature was able to predict the response to chemotherapy.

**Conclusions:**

In conclusion, we identified four different subtypes of breast cancer with significant differences in the immune microenvironment and pathways. We elucidated prognostic biomarkers associated with breast cancer and constructed a prognostic model involving four m7GRGs. In addition, we predicted the candidate drugs related to breast cancer based on the prognosis model.

**Supplementary Information:**

The online version contains supplementary material available at 10.1186/s12885-023-11012-z.

## Introduction

Breast cancer is a malignant tumour that seriously threatens women’s life and health. It is estimated that there are about 1.3 million new breast cancer cases worldwide each year, and 450,000 patients die of breast cancer [1]. The aetiology of breast cancer remains unclear but can be roughly divided into genetic, environmental and hormonal factors, although genetic factors occupy a very important part [2–4]. Epidemiological surveys have found that 5% to 10% of breast cancers are familial and are related to genetic mutations inherited from a parent [5]. At present, the treatment of breast cancer is mainly based on clinical staging, pathological typing and molecular typing. However, breast cancer is a highly heterogeneous disease [6, 7], and the traditional clinicopathological diagnostic approach to breast cancer has been associated with low accuracy and poor specificity. Therefore, more in-depth studies of the molecular mechanisms underlying the progression of breast cancer are needed.

Epigenetics is a process that does not involve alterations in the gene sequence but heritable changes in gene expression and modification. In particular, epigenetic methylation by 7-methylguanosine (m7G) has attracted widespread attention. During transcription initiation, m7G is co-transcribed onto the 5' cap [8]. This cap modification stabilises transcripts, prevents exonucleolytic degradation and regulates nearly every stage of the mRNA life cycle, including transcription elongation, pre-mRNA splicing, polyadenylation, nuclear export and translation. In addition to being part of the cap structure, m7G is also present inside tRNA and rRNA [9], while internal m7G modifications affect RNA processing and function and are believed to be involved in human diseases, especially cancer. m7G modification participates in the occurrence and progression of cancer by regulating the metabolism of various RNA molecules, as well as the expression of oncogenes and tumour suppressor genes [10]. Multiple studies have shown that the regulatory factors of m7G can effectively predict tumour prognosis and the immune therapy response [11]. Indeed, based on m7G-related genes (m7GRGs), Li et al. identified prognostic biomarkers related to gastric cancer [12]. Therefore, the study of m7GRGs may provide promising potential biomarkers for the diagnosis, prognosis and treatment of breast cancer [13].

Current evidence suggests that the tumour immune microenvironment (TIME) plays an important role in cancer occurrence, development, invasion and metastasis. The TIME includes immune cells in various tumour microenvironments (such as T and B lymphocytes), stromal cells and tumour cells, as well as their corresponding expressed and secreted immune molecules [14, 15]. Their interaction has a dual effect on breast cancer: immune effector cells (such as CD8 + cytotoxic T cells) and molecules inhibit tumour cell growth and proliferation through different pathways, while immunosuppressive cells (such as regulatory T cells) and factors (such as IL-10) and inhibitors expressed or secreted by tumour cells inhibit immune responses through different pathways and participate in tumour escape and promote tumour occurrence, development and metastasis [16]. m7G regulates immune responses by participating in cellular processes such as immune cell development, differentiation, activation, migration and polarisation and is involved in cancer progression [17]. Research has confirmed that RNA methylation (such as m7G) can regulate RNA immunogenicity and innate immune components in the tumour immune system and affect the innate immunity of the tumour body [18]. Huang et al., through pan-cancer analysis of m7GRGs, found that m7G had an excellent ability to predict prognosis and the immunotherapy response, which may lead to innovative biomarkers for cancer immunotherapy and prognosis [19].

With developments in bioinformatics, researchers can analyse and interpret biological data with specific algorithms and software [20, 21]. To investigate the function of m7G modification in breast cancer, we explored the expression, copy number variation (CNV) incidence and prognostic value of 24 m7GRGs in breast cancer. We then performed consensus clustering and divided tumour samples into four subtypes with significantly different immune cell infiltration and stromal scores based on the expression of the 24 m7GRGs in the TCGA-BRCA dataset. Differentially expressed genes (DEGs) between subtypes were mainly enriched in immune-related pathways such as TNF signalling pathway and Salmonella infection. Multivariate Cox regression analysis was performed based on 21 prognostic-related m7G genes, and four genes (AGO2, EIF4E3, DCPS and EIF4E) were identified for constructing the prediction model. The samples were divided into high- and low-risk groups according to the median risk score, and survival analysis revealed a significant survival difference between the two groups. The four m7GRGs were able to independently predict the prognosis of BRCA patients. Based on the risk scores of the prognostic model and clinical factors, we constructed a nomogram model to predict the prognosis of breast cancer patients. Finally, we explored the association of m7GRG-related prognostic models with tumour mutational burden (TMB) and drug sensitivity. Accordingly, we report a hitherto undocumented m7G gene signature that can help to improve the prognosis of breast cancer patients in clinical practice.

## Materials and methods

### Datasets

Gene expression profiling data of breast cancer patients were obtained from two independent patient cohorts, The Cancer Genome Atlas (TCGA) dataset TCGA-BRCA and the Gene Expression Omnibus (GEO) dataset GSE1456. The TCGA-BRCA cohort included 113 normal samples and 1113 breast cancer samples. We used the ‘limma’ package to normalise gene expression data and remove genes with a mean expression level of 0. Somatic data for breast cancer were also obtained from TCGA. Somatic data were preprocessed through Perl-related code. TCGA-BRCA data were annotated with gene names using GENCODE22 annotation files, and TCGA-BRCA patient survival data and clinical data were obtained from UCSC Xena, including survival time, survival status, age, tumour stage and sex. CNV data for breast cancer were also obtained from UCSC Xena. In our study, patient samples with clinical information and a survival time greater than 30 days were retained. Finally, 1023 patient samples were included from TCGA-BRCA and 159 from GSE1456; TCGA-BRCA was used for model construction, whereas GSE1456 was used for model validation.

### Mutation analysis and prognostic analysis of m7GRGs

m7GRGs (DCP2, IFIT5, EIF3D, EIF4G3, NSUN2, GEMIN5, AGO2, NUDT10, EIF4E, EIF4E2, NCBP2, NUDT11, NUDT3, NCBP1, METTL1, LARP1, NUDT4, EIF4E3, SNUPN, WDR4, LSM1, NUDT16, DCPS and CYFIP1) were obtained from the existing literature [22] and related gene sets: GOMF_m7G_5_PPPN_DIPHOSPHATASE_ACTIVITY, GOMF_RNA_CAP_BINDING and OMF_RNA_7_METHYLGUANOSINE_CAP_BINDING. We extracted the expression of 24 m7GRGs from the TCGA-BRCA queue and then used the ‘limma’ algorithm to analyse the difference between normal samples and breast cancer samples. m7GRGs with a *p*-value less than 0.05 were considered differentially expressed. Next, we explored the CNV incidence of the 24 m7GRGs and mapped their altered locations on 23 chromosomes using the ‘RCircos’ package. To elucidate the correlation of the 24 m7GRGs with breast cancer patients’ prognosis, we used the ‘igraph’, ‘psych’, ‘reshape2’ and ‘RColorBrewer’ packages to plot the prognosis-related network of the 24 m7GRGs.

### Consensus clustering of m7GRGs and functional enrichment analysis

Based on the expression profile data of these 24 genes, the K-means clustering algorithm of the ‘ConsensusClusterPlus’ package was used to perform consensus clustering on TCGA-BRCA patients to obtain breast cancer subtypes. The clustering was repeated 1000 times to ensure the accuracy and stability of the results. The optimal number (K-value) of breast cancer subtypes was calculated using the cumulative distribution function (CDF) and a consensus heatmap.

To explore the differences between different breast cancer subtypes, we used the ‘limma’ package to identify DEGs across the breast cancer subtypes, with thresholds set at *p* < 0.05 and |logFold Change|> 1. To identify the enriched pathways among the different subtypes, we conducted Gene Ontology (GO) and Kyoto Encyclopaedia of Genes and Genomes (KEGG) enrichment analyses [23, 24] for DEGs between subtypes and visualised the top 5 pathways with the most significant enrichment results. Using the gene set enrichment analysis (c2.cp.kegg.v7.5.1.symbols.gmt of the MSigDB reference gene set), the top 10 pathways were visualised. The above enrichment analysis was conducted using the ‘clusterProfiler’ package.

### Comparison of immune cell infiltration among m7G patterns

To explore the extent of immune cell infiltration among different subtypes, we used the ‘IOBR’ package to assess immune cell infiltration with the ESTIMATE algorithm and the CIBERSORT algorithm in the TCGA-BRCA dataset and obtained the immune cell infiltration of each sample in both algorithms. The indicators evaluated by the ESTIMATE algorithm include immune score, stromal score and tumour purity. The CIBERSORT algorithm enables quantification of the relative abundance of 22 types of immune cells. The abundance differences of 22 immune cells among the different breast cancer subtypes were calculated using the Wilcoxon test. Differences in immune cell infiltration among different subtypes were considered significant at *p* < 0.05.

### Support vector machines and multivariate Cox regression analysis

We used the support vector machine (SVM) algorithm of the ‘e1071’ package to select the prognosis-related features in the TCGA-BRCA expression profiling data of the 24 m7GRGs. Of the 24 prognosis-related genes, 21 were selected and used for survival analysis. Multivariate Cox analysis was used to construct predictive models, and survival analysis was performed using the ‘survival’ package. Subsequently, we validated the model in the GSE1456 dataset, with *p* < 0.05 considered statistically significant. Kaplan–Meier analysis was used to evaluate the difference in survival between high- and low-risk groups. In addition, we integrated the risk scores and clinical features for univariate and multivariate Cox analyses to verify that the risk scores were predictive markers independent of other clinical features and constructed nomograms based on the clinical features associated with prognosis using the ‘rms’ package to more accurately predict patient prognosis. The results of nomogram prediction were verified by calibration curve and ROC curve analysis to ensure the accuracy of the nomogram for predicting the 1-year, 3-year and 5-year survival rates of patients.

### TMB analysis and drug sensitivity prediction

**Fig. 1:**
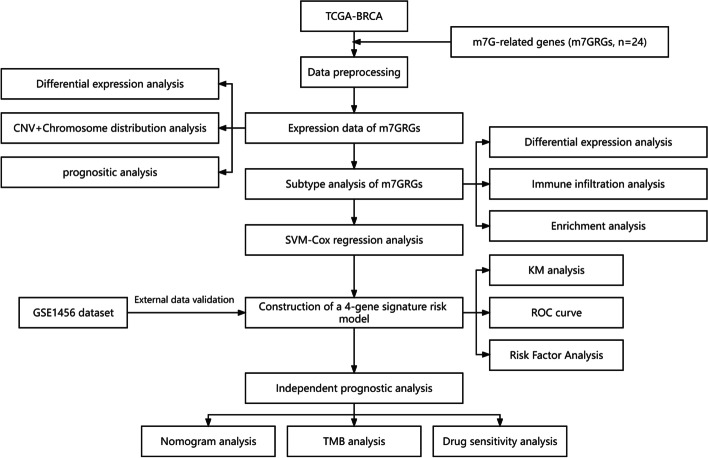
Flowchart of this study

The ‘maftools’ package was used to draw waterfall plots related to TMB. Kaplan–Meier analysis was applied to evaluate the effect of TMB on the prognosis of breast cancer patients. Moreover, we used t-tests to determine differences in TMB levels between the high- and low-risk groups. Spearman's rank correlation was applied to calculate the correlation between TMB and risk score. Then, we estimated each patient's sensitivity to chemotherapeutic drugs using the Genomics of Cancer Drug Sensitivity (GDSC) database. The half-maximal inhibitory concentration (IC_50_) values for high- and low-risk groups were quantified with the ‘pRRophetic’ package. We conducted the Wilcoxon test to calculate the differences in the IC_50_ of the drugs among the breast cancer risk subgroups. The CellMiner database was used to mine sensitive drugs to build a predictive model. In addition, Pearson analysis was used to calculate drugs related to risk scores. The overall flowchart of this study is shown in Fig. 1.

### Cell culture and quantitative real-time polymerase chain reaction

MDA-MB-231, MDA-MB-468, SKBR3 and MCF-10A cells were purchased from Procell Life Science & Technology Co., Ltd. (Wuhan, China). MDA-MB-231 cells were incubated in DMEM culture medium (Gibco, 11,965,092) with 10% foetal bovine serum (FBS). MDA-MB-468 cells were incubated in RPMI 1640 culture medium (Gibco, 11,875,093) with 10% FBS. SKBR3 cells were cultured in special culture medium (Procell, CM-0211) containing McCoy's 5A, 10% FBS and 1% penicillin/streptomycin. MCF-10A was cultured in special culture medium (Procell, CM-0525) containing DMEM/F12, 5% Horse serum, 20 ng/mL EGF, 0.5 μg/mL Hydrocortisone, 10 μg/mL Insulin, 1% NEAA and 1% P/S. Total RNA was isolated with the TRIzol reagent (Invitrogen). Complementary DNA was synthesized from 1 μg total RNA using MightyScript Plus First Strand cDNA Synthesis Master Mix (Sangon Biotech). Quantitative real-time polymerase chain reaction (qRT-PCR) was performed with a PowerUp SYBR Green Master Mix (Thermo Fisher, A25742) after RNA extraction and reverse transcription from all four cell lines. Relative mRNA levels were calculated using the comparative Ct method (ΔCt). The primer sequences are listed in Supplementary Material S1.

## Results

### Landscape of m7GRG expression, genetic variation and prognostic relevance in breast cancer

**Fig. 2:**
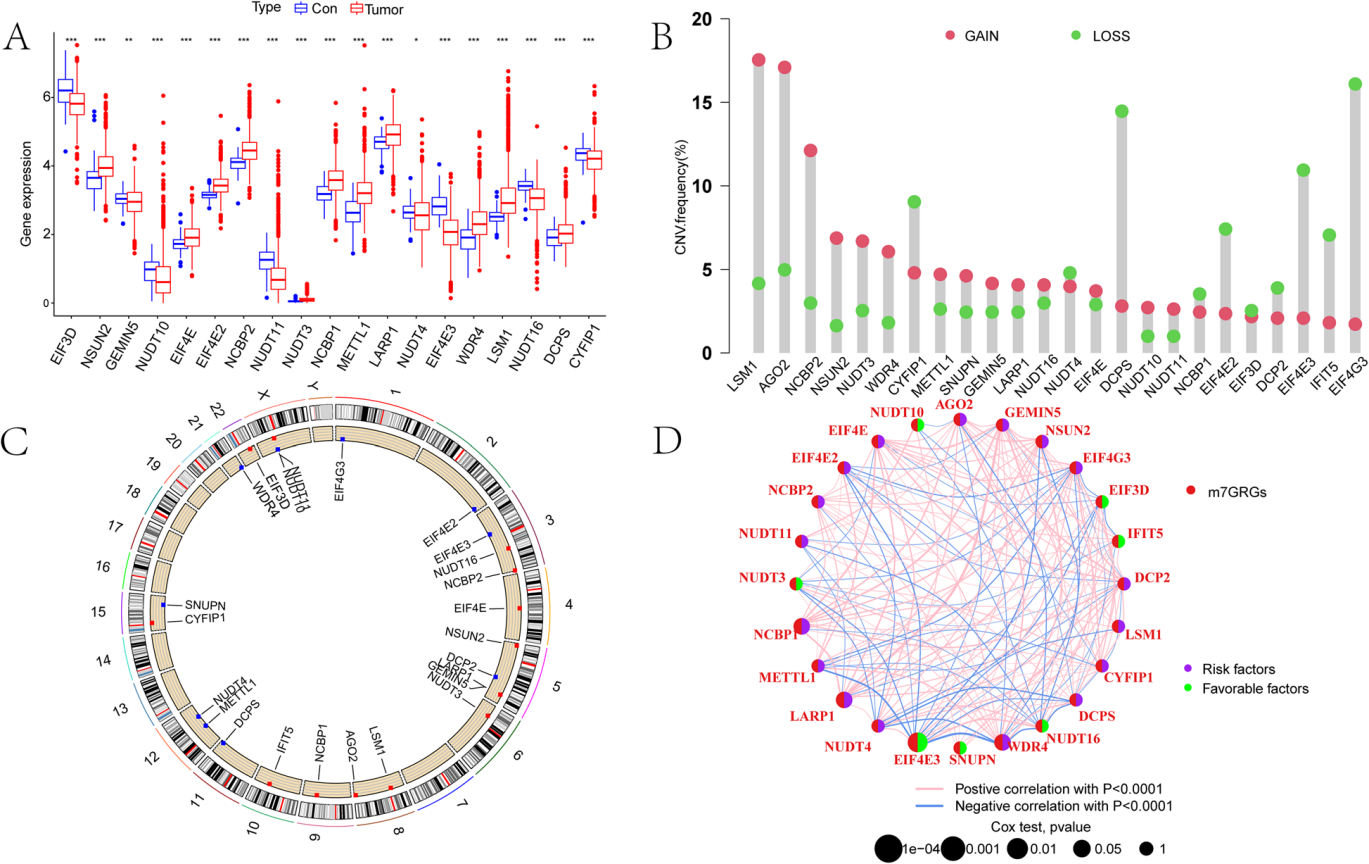
Characteristics and differences of 24 m7GRGs in breast cancer. **A** Expression of the 24 m7GRGs in breast cancer and normal tissues. **B** Mutation frequency and classification of the 24 m7GRGs in breast cancer. **C** Locations of the CNV alterations of the 24 m7GRGs on the 23 chromosomes in the breast cancer cohort. **D** Circos graph for univariate Cox regression analysis, which represents the association of the expression of the 24 m7GRGs with breast cancer prognosis in the TCGA-BRCA cohort. *p < 0.05, **p < 0.01, ***p < 0.001

First, we explored the expression of 24 m7GRGs in breast cancer tissues and normal tissues in the TCGA-BRCA cohort. Overall, 19 m7GRGs were differentially expressed between breast cancer tissues and normal tissues. In particular, the expressions of NSUN2, EIF4E, EIF4E2, NCBP2, NUDT3, NCBP1, LARP1, WDR4 and LSM1 were upregulated (Fig. 2A, Supplementary Material S2). Subsequently, we identified the CNV incidence of the 24 m7GRGs. In the TCGA-BRCA cohort, CNV alterations were found in all 24 m7GRGs. In addition, more than half of the m7GRGs had copy number amplification, while CYFIP1, NUDT4, DCPS, NCBP1, EIF3D, DCP2, EIF4E3, IFIT5 and EIF4G3 had CNV deletions (Fig. 2B). Figure 2C shows the locations of the CNV alterations of the 24 m7GRGs on chromosomes. Finally, to explore the association of the 24 m7GRGs with breast cancer prognosis, we mapped the network of interactions among the 24 m7GRGs and the effect of their expression on breast cancer prognosis. The results showed that 17 m7GRGs were risk factors and significantly impacted breast cancer prognosis, whereas NUDT10, NUDT3, EIF4E3, SNUPN, NUDT16, IFIT5 and EIF3D were favourable prognostic factors (Fig. 2D). In addition, based on the MethSurv website (https://biit.cs.ut.ee/methsurv/), we explored the changes in DNA methylation in the 24 m7GRGs (Supplementary Material S3).

### Identification of breast cancer classification patterns mediated by the 24 m7GRGs

**Fig. 3:**
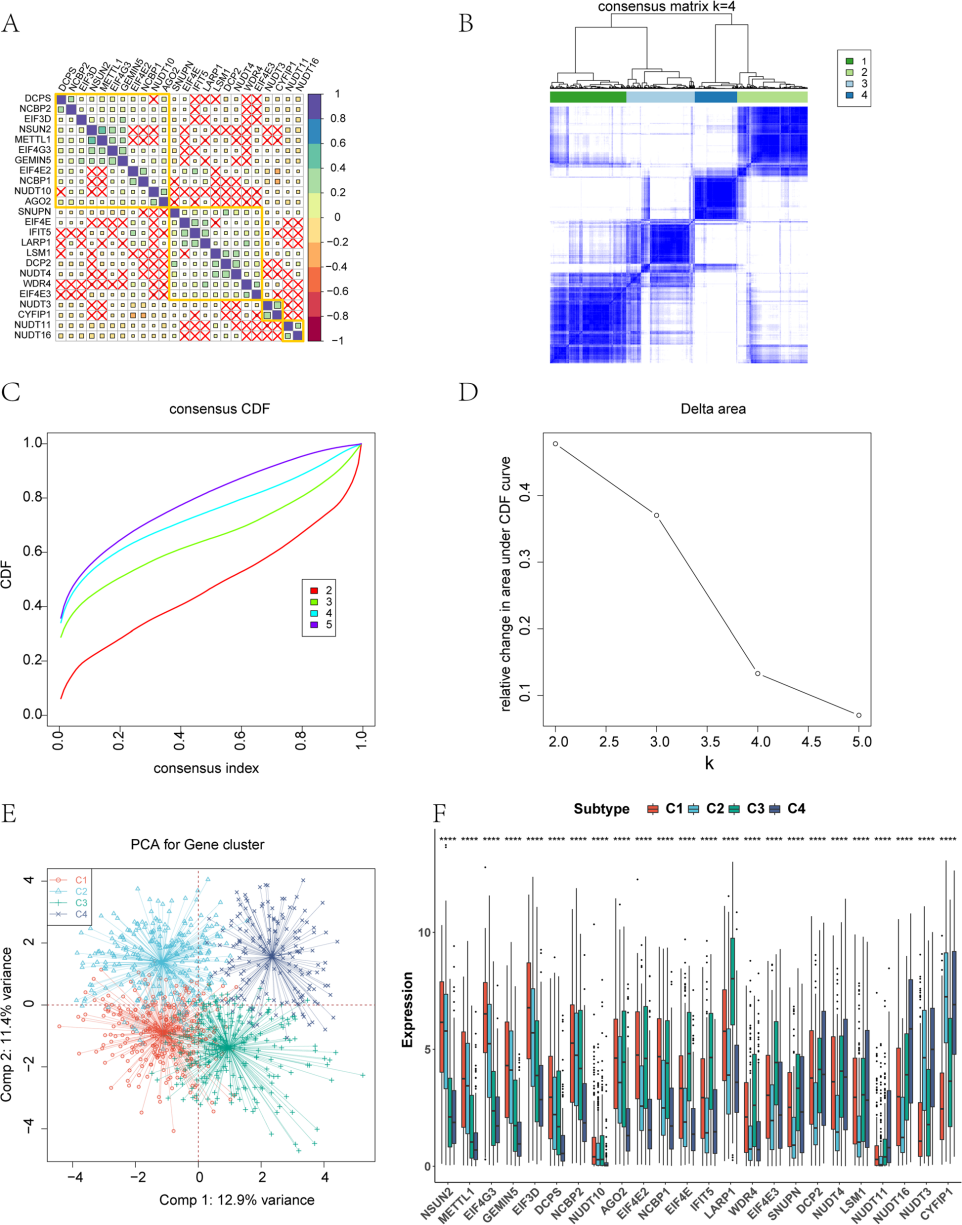
Breast cancer subgroups related by the 24 m7GRGs. **A** Correlations among the 24 m7GRGs in breast cancer. **B** Consensus score matrix of breast cancer samples when k = 4 in TCGA-BRCA cohorts. **C** Census CDF curves for the TCGA-BRCA cohort. **D** The delta area under the CDF curve shows the change in cumulative risk with increases in the consensus clustering matrixes and demonstrated that three clusters were optimal (k = 4). **E** PCA plots for four clusters in the TCGA-BRCA cohort. **F** Expression of the 24 m7GRGs among the four breast cancer subtypes. *p < 0.05, **p < 0.01, ***p < 0.001

Correlation analysis of the 24 m7GRGs in the TCGA-BRCA dataset showed a close correlation among these genes (Fig. 3A) (*p* < 0.05). Based on the expression of the 24 m7GRGs, we used consensus clustering analysis to classify 1023 breast cancer samples into four subtypes: C1, C2, C3 and C4 (Fig. 3B-D, Supplementary Material S4). Principal component analysis (PCA) revealed that the four subtypes were clearly separated (Fig. 3E), indicating there were significant differences in expression profiles among the different subtypes. At the same time, the expression levels of the 24 genes were significantly different among the four subtypes (Fig. 3F), showing that our typing results had good stability and accuracy.

**Fig. 4:**
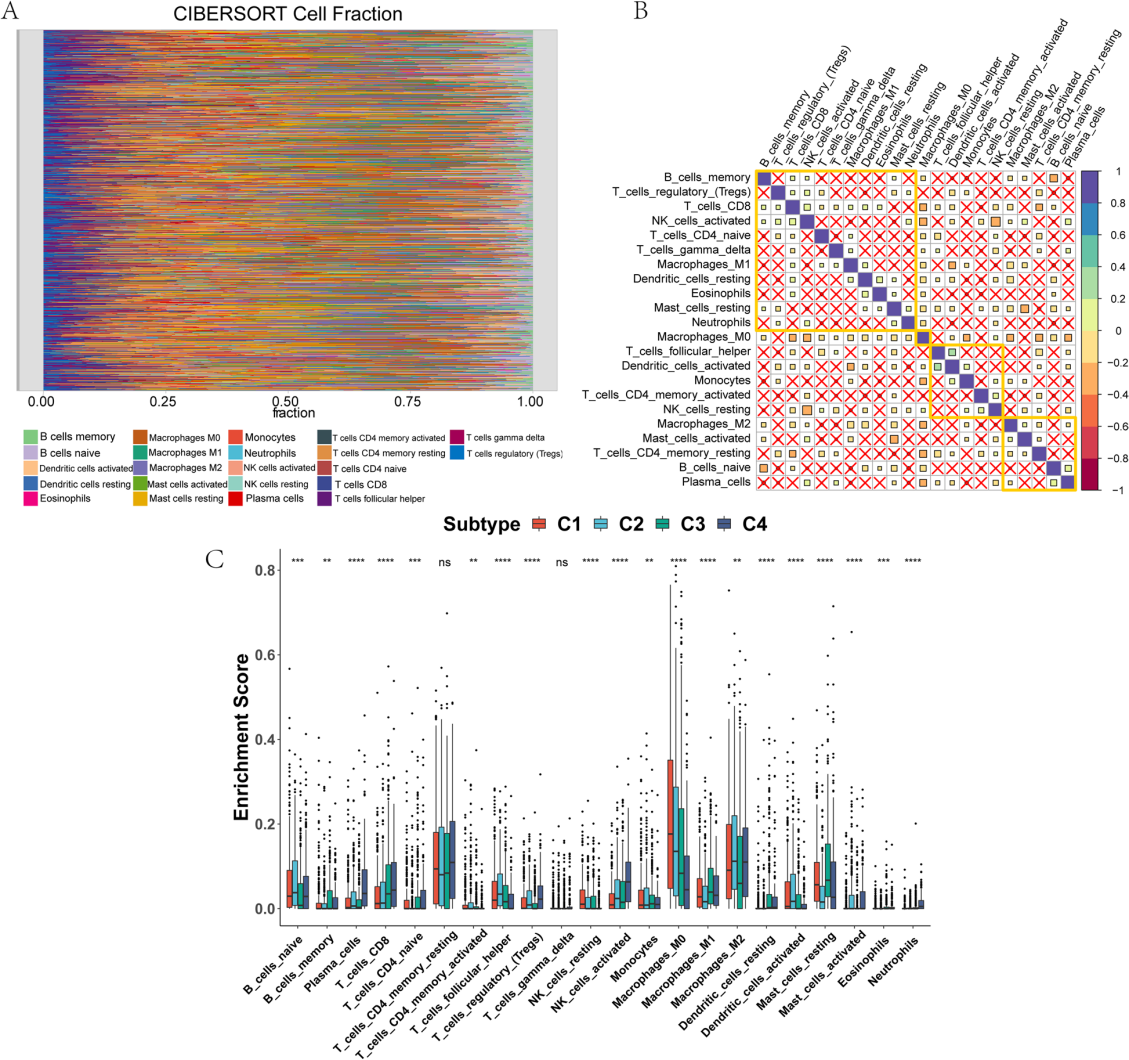
Landscape of immune infiltration among the four breast cancer subtypes. **A** Distribution of 22 immune cells in 1023 breast cancer samples. **B** Correlations among 22 immune cells in the TCGA-BRCA cohort. **C** Differences in the infiltration levels of 22 immune cells among the four breast cancer subtypes. *p < 0.05, **p < 0.01, ***p < 0.001. ns means no significance

**Fig. 5:**
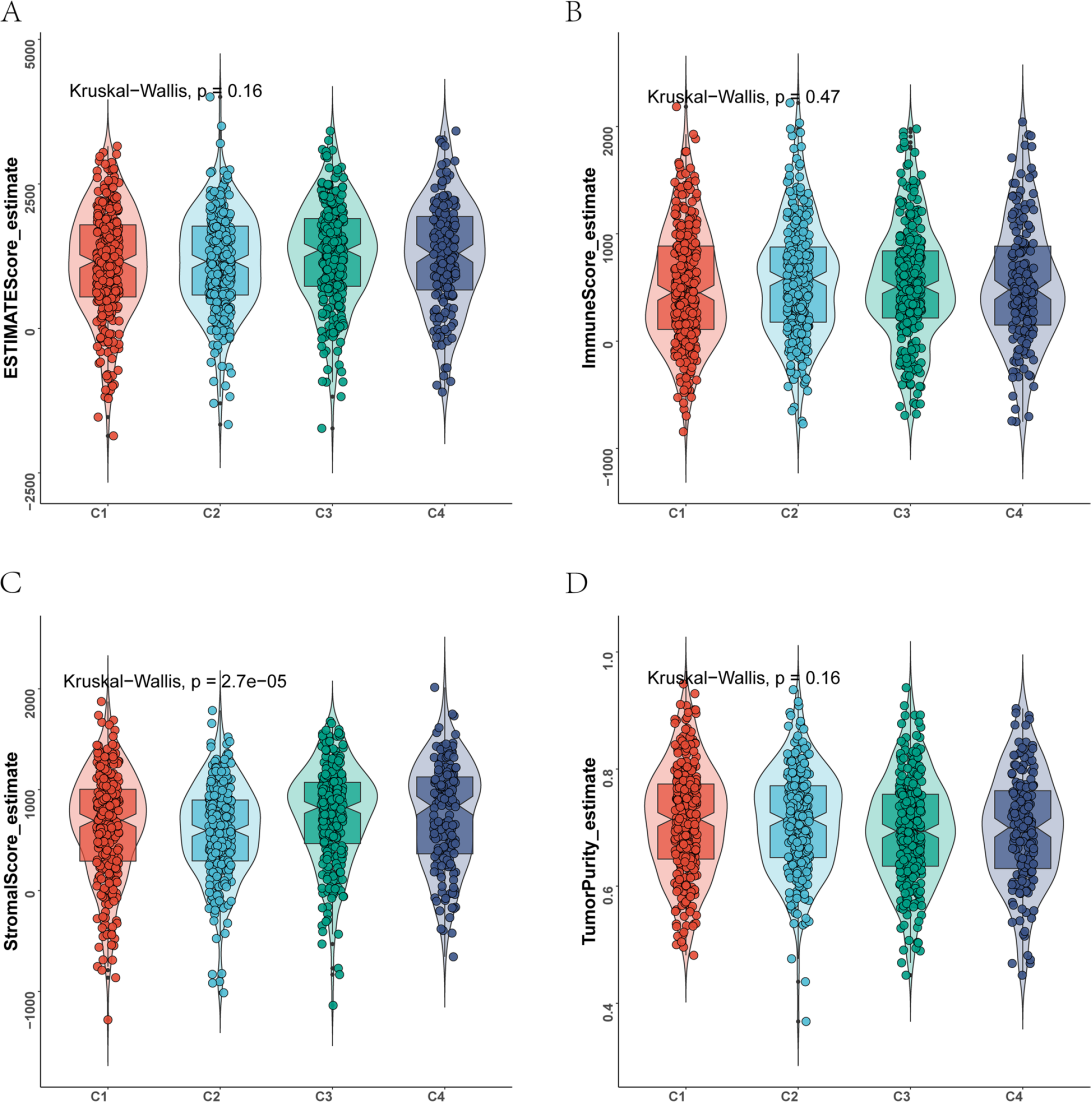
Results of ESTIMATE analysis among the four breast cancer subtypes. **A** ESTIMATEScore for the four breast cancer subtypes. **B** ImmuneScore for the four breast cancer subtypes. **C** StromalScore for the four breast cancer subtypes. **D** TumorPurity for the four breast cancer subtypes. A *p*-value less than 0.05 was considered statistically significant

To explore differences in immune infiltration levels among the four breast cancer subtypes, we performed immune infiltration analysis on the four subtypes. The CIBERSORT results showed that the infiltration degree of the 22 types of immune cells differed among the different samples (Fig. 4A), and a significant correlation was present between T cells and natural killer cells (Fig. 4B), indicating a synergistic effect between these cells in BRCA patients. Moreover, the expression of most immune cells differed significantly among different subtypes. Specifically, Plasma_cells, T_cells_CD8, T_cells_follicular_helper, T_cells_regulatory_(Tregs), NK_cells_resting, NK_cells_activated, Macrophages_M0, Macrophages_M1, Dendritic_cells_resting, Dendritic_cells_activated, Mast_cells_resting, Mast_cells_activated and Neutrophils exhibited significant differences in immune infiltration abundance among the four breast cancer subtypes (Fig. 4C). The ESTIMATE results showed that the stromal score significantly differed among the subtypes (*p* < 0.05) (Fig. 5A-D), indicating a significant difference in stromal cell composition among subtypes.

### Functional enrichment analysis among breast cancer subtypes

**Fig. 6:**
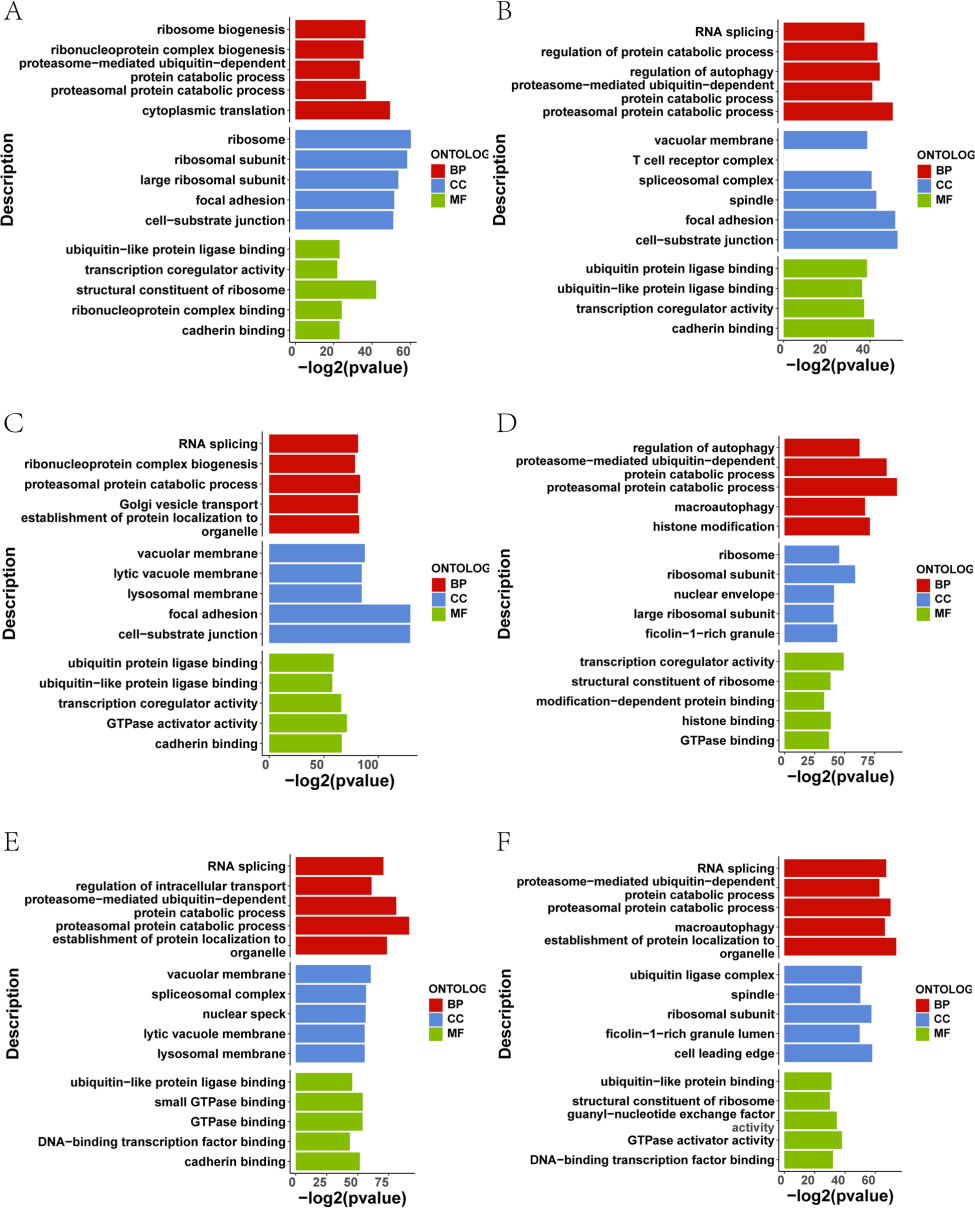
GO enrichment analysis between the four breast cancer subtypes. **A** Functional pathways enriched between C1 and C2. **B** Functional pathways enriched between C1 and C3. **C** Functional pathways enriched between C1 and C4. **D** Functional pathways enriched between C2 and C3. **E** Functional pathways enriched between C2 and C4. **F** Functional pathways enriched between C3 and C4

**Fig. 7:**
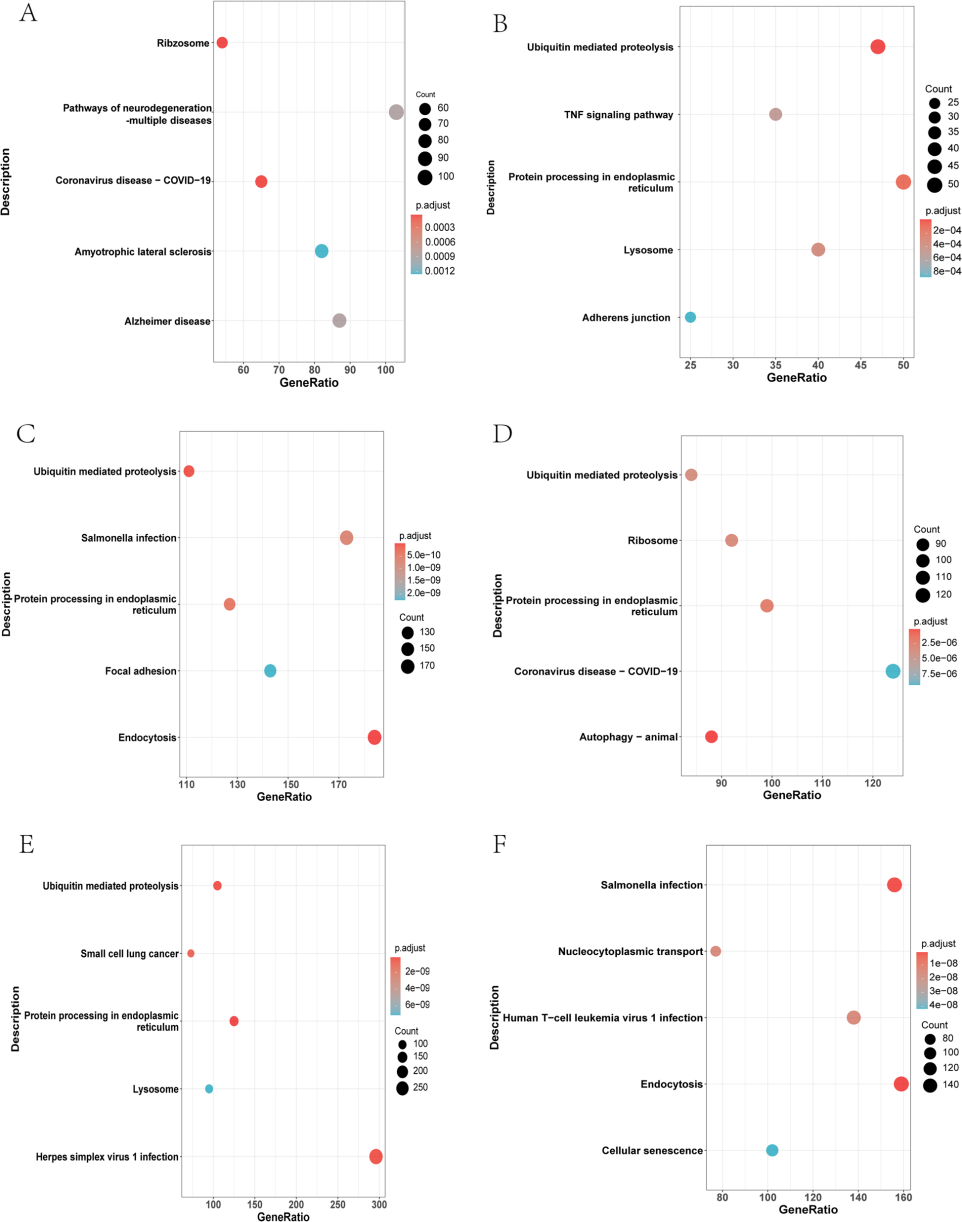
KEGG enrichment analysis between the four breast cancer subtypes. **A** Pathways enriched between C1 and C2. **B** Pathways enriched between C1 and C3. **C** Pathways enriched between C1 and C4. **D** Pathways enriched between C2 and C3. **E** Pathways enriched between C2 and C4. **F** Pathways enriched between C3 and C4. A *p*-value less than 0.05 was considered statistically significant

**Fig. 8:**
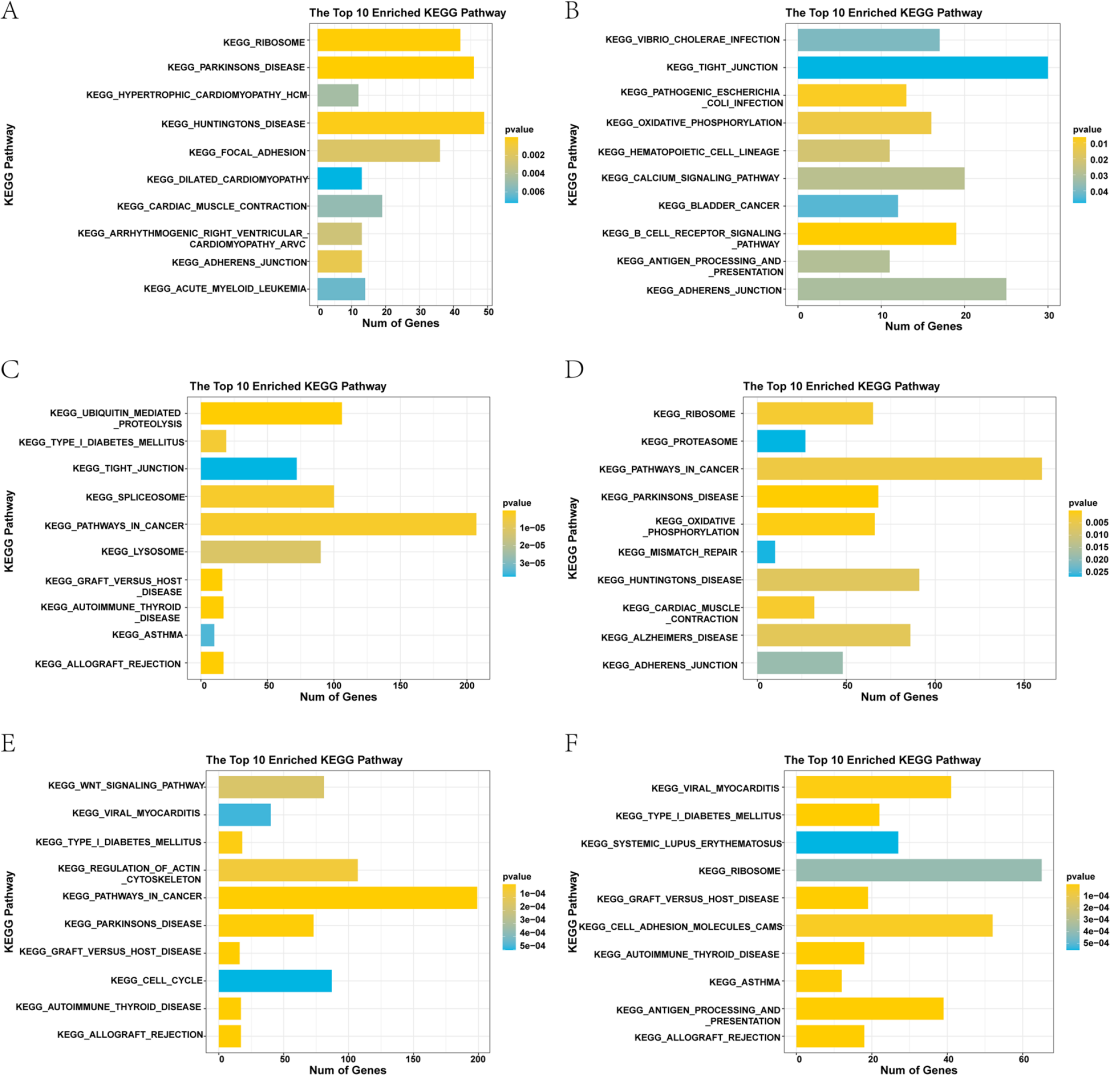
GSEA enrichment analysis between the four breast cancer subtypes. **A** Pathways enriched between C1 and C2. **B** Pathways enriched between C1 and C3. **C** Pathways enriched between C1 and C4. **D** Pathways enriched between C2 and C3. **E** Pathways enriched between C2 and C4. **F** Pathways enriched between C3 and C4. A *p*-value less than 0.05 was considered statistically significant

To identify the biological functions and pathways involved in the DEGs among the four breast cancer subtypes in breast cancer, we performed enrichment analysis of DEGs between the different breast cancer subtypes (C1 vs C2, C1 vs C3, C1 vs C4, C2 vs C3, C2 vs C4, C3 vs C4). GO enrichment analysis (Fig. 6A-F) revealed significant enrichment in ribosome biogenesis, RNA splicing and macrophage pathways. KEGG enrichment analysis results (Fig. 7A-F) showed that the DEGs were mainly enriched in Ribosome, TNF signalling pathway and Salmonella infection. The GSEA results (Fig. 8A-F) showed significant enrichment in KEGG_RIBOSOME, KEGG_VIBRIO_CHOLERAE_INFECTION, KEGG_PROTEASOME and other pathways. These results suggest that m7GRGs may be involved in numerous biological processes and pathways related to immunity.

### Construction of a prognostic model related to m7GRGs

**Fig. 9:**
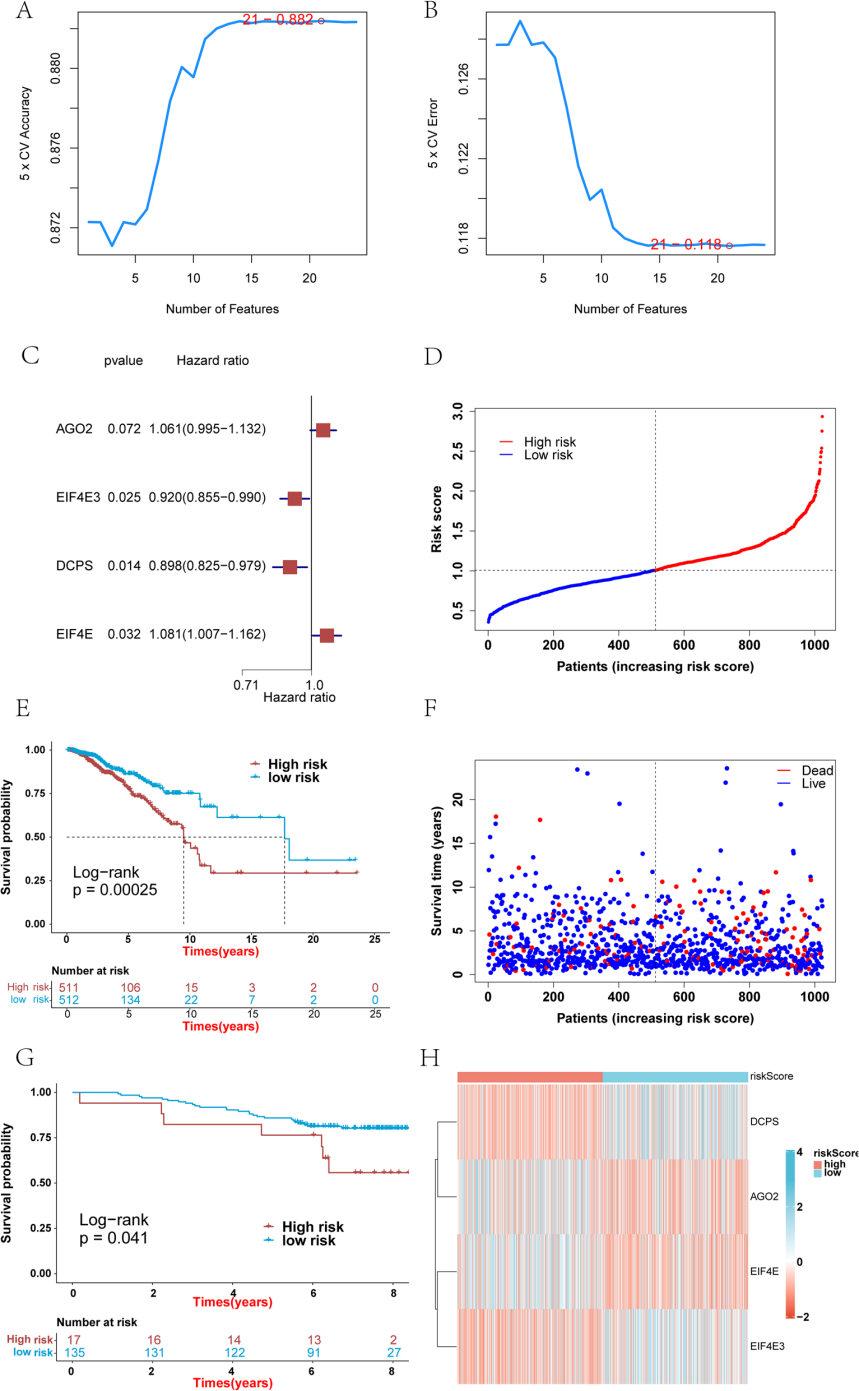
Construction of a prognostic model involving four m7GRGs. **A** and **B** Accuracy and error of fivefold cross-validation (CV) in SVM analysis. **C** Forest plot for hazard ratios of the four m7GRGs. **D** Distribution of patients in the TCGA-BRCA based on the risk score. **E** Kaplan–Meier curves for breast cancer patients in the high-/low-risk groups in the TCGA-BRCA. **F** Survival status for each patient in the TCGA-BRCA. **G** Kaplan–Meier curves for breast cancer patients in the high-/low-risk group in GSE1456. **H** Heatmap for the connections between the expression of the four m7GRGs and the risk groups in the TCGA-BRCA. A *p*-value less than 0.05 was considered statistically significant

**Fig. 10:**
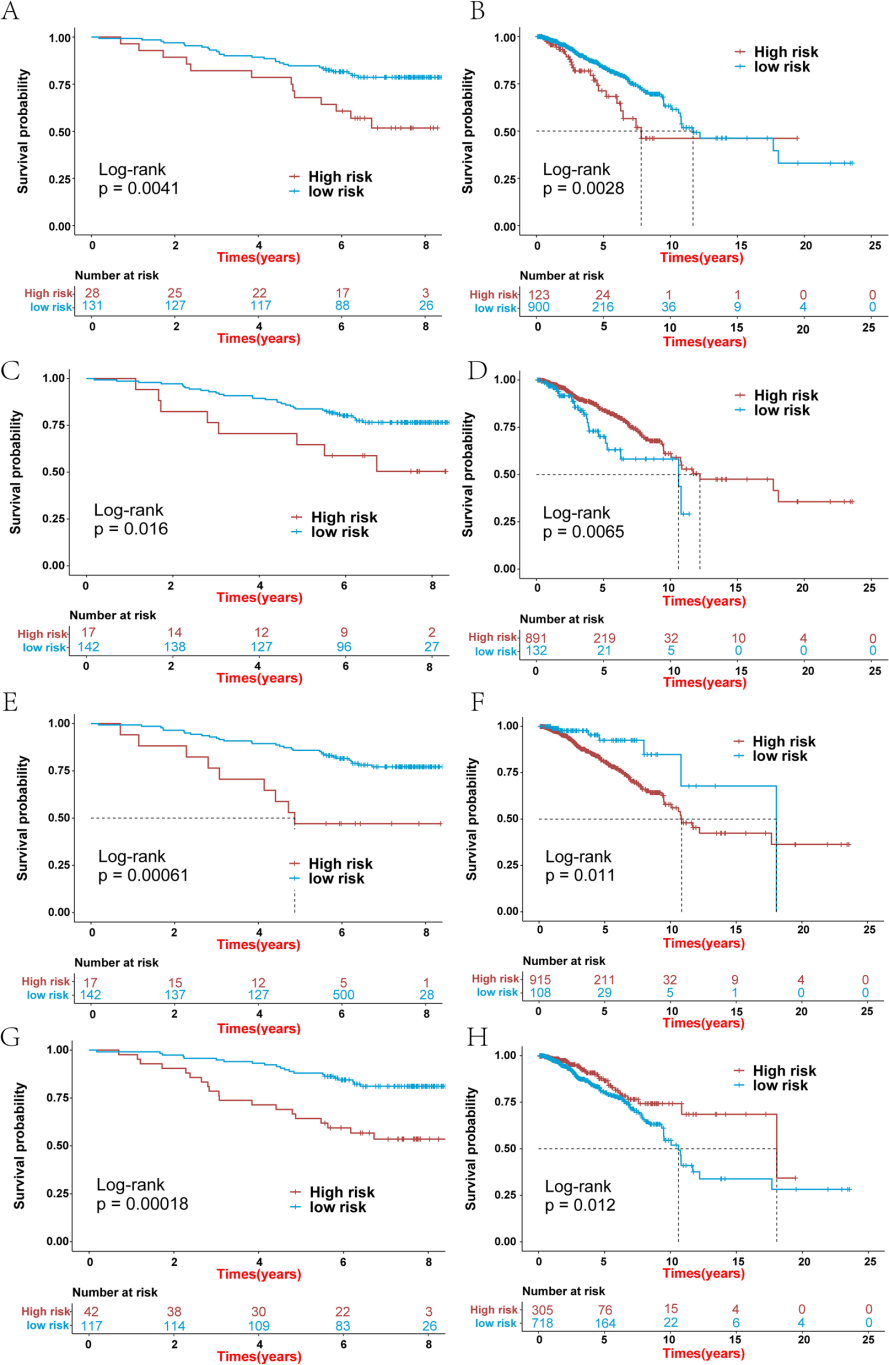
Kaplan–Meier curve of the four m7GRGs (AGO2, EIF4E3, DCPS and EIF4E) in breast cancer. The overall survival curve of AGO2 (**A**), EIF4E3 (**C**), DCPS (**E**) and EIF4E (**G**) in breast cancer patients in the high-/low-expression groups (GSE1456). Overall survival curve of AGO2 (**B**), EIF4E3 (**D**), DCPS (**F**) and EIF4E (**H**) in breast cancer patients in the high-/low-expression groups (TCGA-BRCA). A *p*-value less than 0.05 was considered statistically significant

To screen for m7GRGs associated with breast cancer patient survival time, we performed SVM analysis on the 24 m7GRGs. We obtained 21 m7GRGs associated with prognosis using the SVM (Fig. 9A and B) and subjected them to multivariate Cox regression analysis. Four genes (AGO2, EIF4E3, DCPS and EIF4E) were identified and used to construct a prediction model (Fig. 9C) that could provide the risk score of each sample in the TCGA-BRCA. Finally, the samples were divided into high- and low-risk groups according to the median risk score. The survival analysis showed a significant difference between the high- and low-risk groups (Fig. 9E). The difference in survival was also significant between high- and low-risk group samples in the validation set GSE1456 (Fig. 9G). The high-risk group was correlated with a lower survival rate (Fig. 9D, F). The expression levels of the four genes used to construct the predictive model were also significantly different between the high- and low-risk groups (Fig. 9H). Single-gene survival analysis was performed in the GSE1456 (Fig. 10A, C, E and G) and TCGA-BRCA (Fig. 10B, D, F and H) datasets based on the expression levels of the four genes. The results showed significant differences in survival between the high- and low-expression groups, further indicating that these four genes were significantly correlated with the prognosis of BRCA. To more deeply evaluate the clinical impact of AGO2, EIF4E3 and EIF4E in breast cancer, we used the Kaplan–Meier plotter database to plot the DMFS (distant metastasis-free survival)-related Kaplan–Meier curves of AGO2, EIF4E3 and EIF4E. The results showed that the DMFS rates of AGO2 and EIF4E3 were significantly different, which may be related to the metastasis of breast cancer (Supplementary Material S5).

### Assessment of four m7GRGs as independent BRCA prognostic factors

**Fig. 11:**
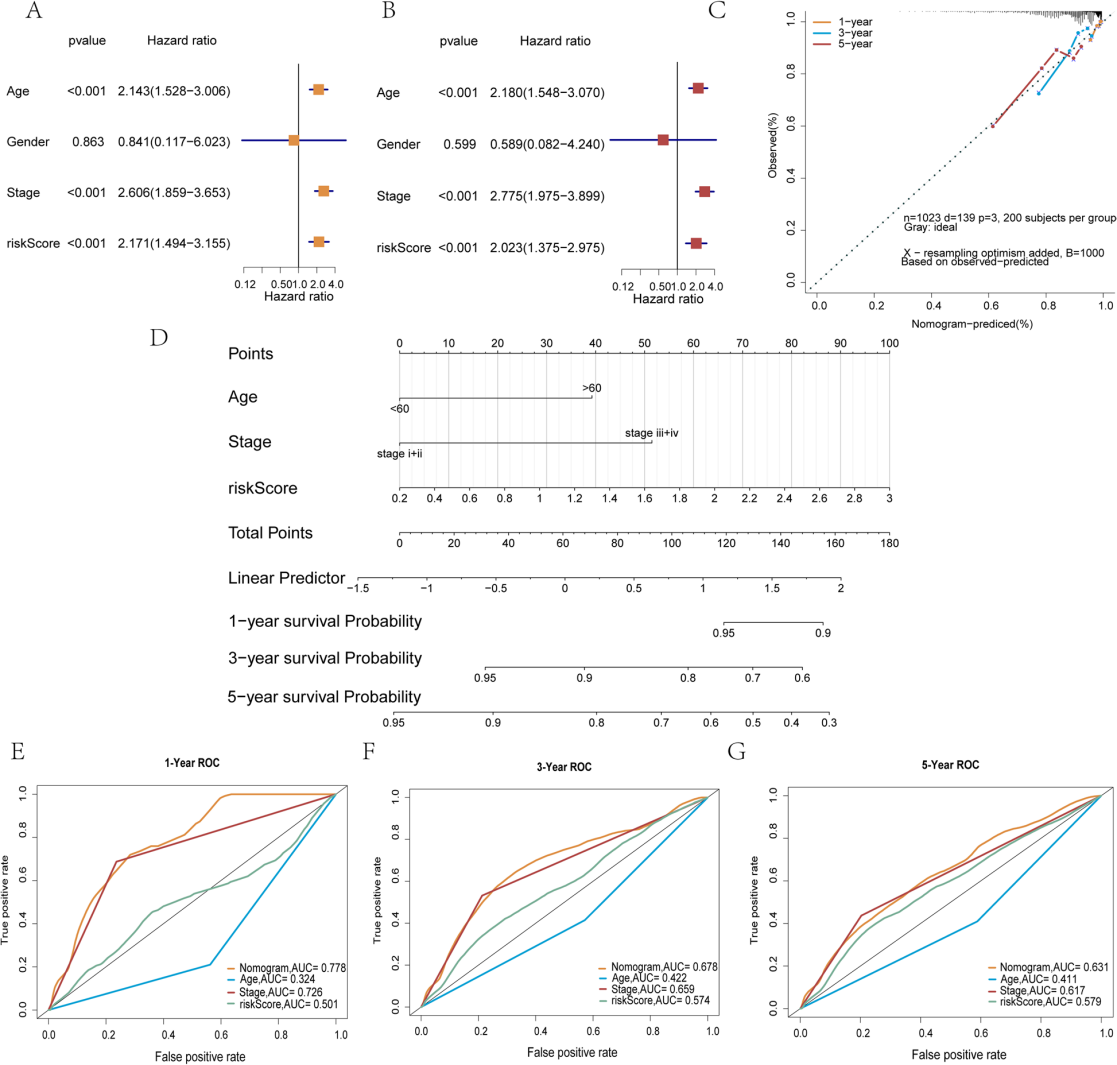
Construction of the nomogram. **A** and **B** Forest plot for hazard ratios of clinical parameters in breast cancer. **C** 1-, 3- and 5-year calibration curves for nomograms. The dashed diagonal line represents the ideal nomogram. **D** Nomogram to predict the 1-, 3- and 5-year overall survival (OS) rates of breast cancer patients. 1- (**E**), 3- (**F**) and 5-year (**G**) ROC curves for nomograms

To assess the prognostic value of the risk score, we performed a prognostic analysis of risk scores and other clinical characteristics in the TCGA-BRCA cohort. Univariate Cox regression analysis (Fig. 11A) revealed that age, tumour stage and risk score were significantly associated with prognosis (*p* < 0.05). Further multivariate Cox regression analysis confirmed (Fig. 11B) that age, tumour stage and risk score were independent predictors of prognosis (*p* < 0.05). Therefore, we constructed a nomogram (Fig. 11D) based on these three clinical characteristics to predict the 1-, 3- and 5-year survival rates of patients. Calibration and ROC curves were used to validate the accuracy of the nomogram in predicting survival time in breast cancer patients. The calibration curve results showed that the predicted values at 1, 3 and 5 years slightly deviated from the diagonal line (Fig. 11C), indicating that the nomogram can satisfactorily predict the prognosis of breast cancer patients compared to the ideal model. We conducted ROC curve analysis on the nomogram for 1 year, 3 years, and 5 years (Fig. 11E-G). The ROC curve showed that the AUC values at 1 year (AUC = 0.778, Fig. 11F) and 3 years (AUC = 0.678, Fig. 11G) were higher. Furthermore, we calculated the C-index of the column chart model, and the results further confirmed its high predictive accuracy (Supplementary Material S6). Overall, both methods demonstrated that the nomogram has better accuracy for predicting patient prognosis.

### TMB and drug sensitivity analysis of m7GRGs

**Fig. 12:**
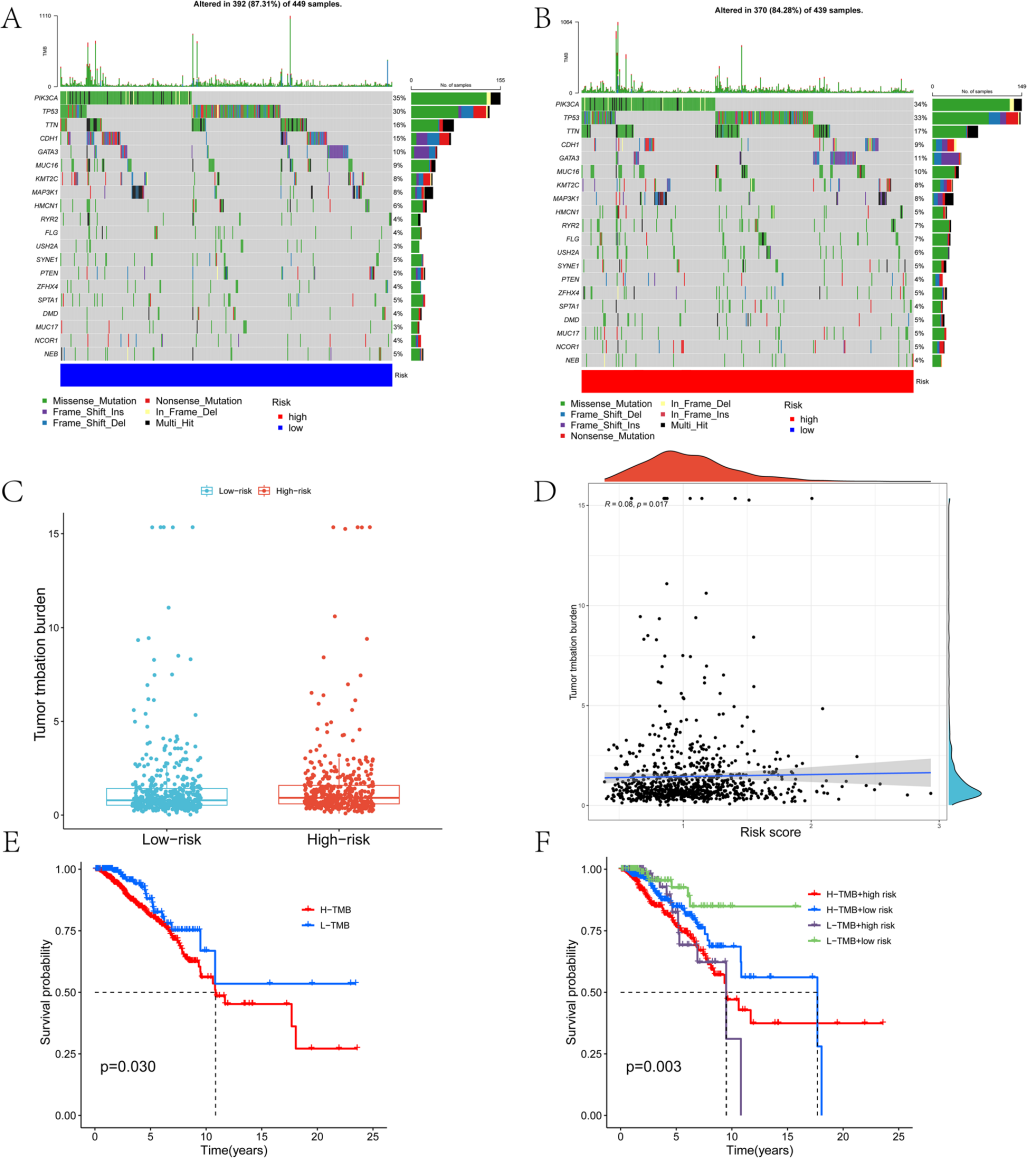
Tumour somatic mutation analysis between the high- and low-risk scores. **A** and **B** are waterfall charts for high- and low-risk groups, respectively. **C** Boxplot of TMB scores in the high- and low-risk groups. **D** Scatter plot of the correlation between TMB score and risk score. **E** OS analysis of BRCA patients in the high- and low-TMB groups. **F** OS analysis of patients with high/low mutational burden in the high-risk group and patients with high/low mutational burden in the low-risk group

Because genetic mutations are an essential cause of the development of BRCA-related breast cancer, we explored differences in the distribution of somatic mutations between high- and low-risk populations. The 20 most frequently mutated genes for these two groups are shown in Fig. 12A and B, respectively. There were no significant differences in the mutation frequency of the top 20 genes between the high- and low-risk groups. The expression levels of risk scores were statistically different between the low- and high-TMB groups (Fig. 12C). The correlation between the mutational burden of BRCA and the population risk score was weak but statistically significant (Fig. 12D). The Kaplan–Meier curve of OS indicated that the OS of the patients in the high-TMB group was significantly lower than that of the patients in the low-TMB group (*p* = 0.03) (Fig. 12E). In addition, the OS of patients with high/low mutational burden in the high-risk group was statistically different from that in the low-risk group with high/low mutational burden (*p* = 0.03) (Fig. 12F). Therefore, high TMB may be an important factor leading to poor OS in breast cancer patients.

**Fig. 13:**
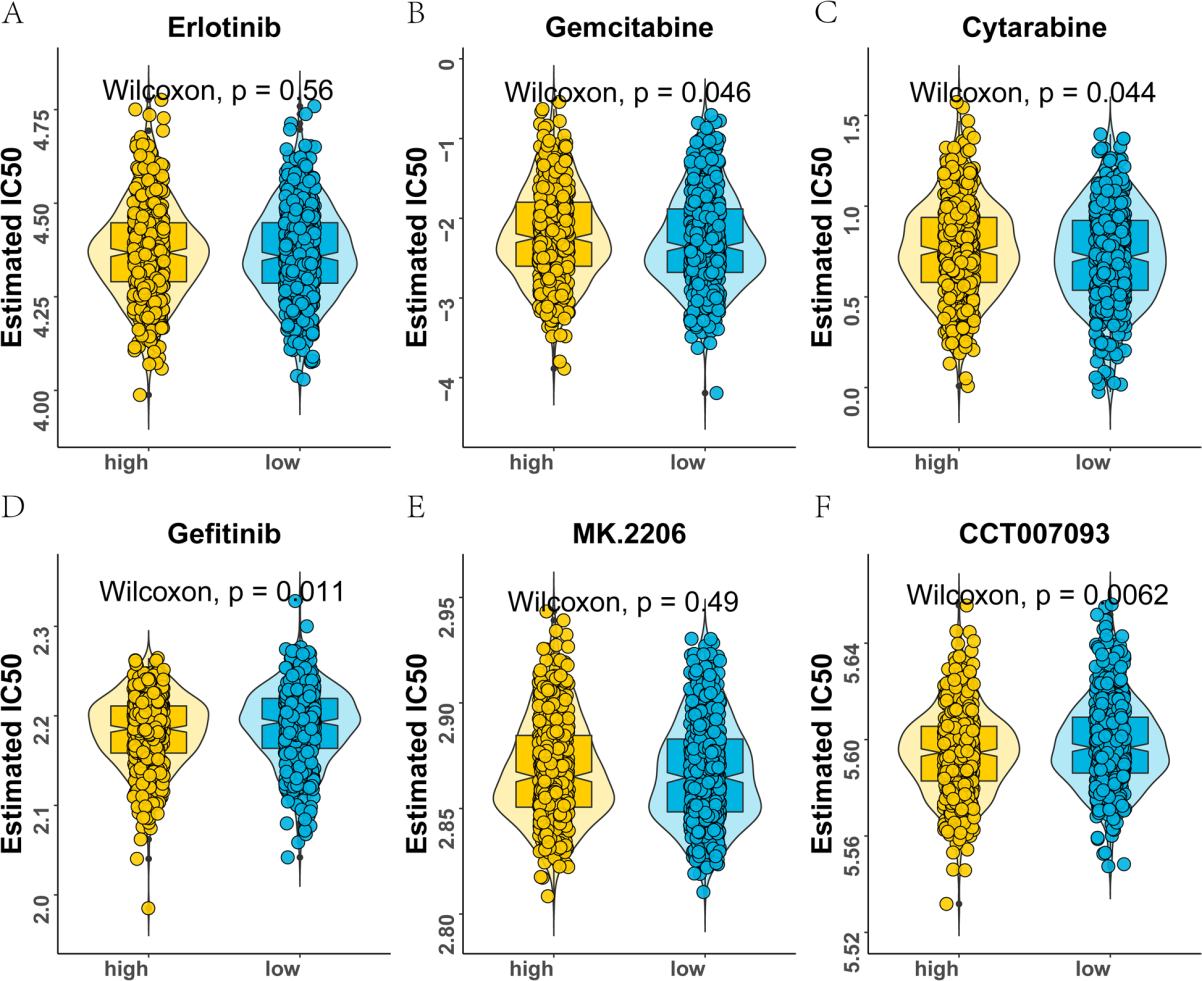
Differences in drug sensitivity between high- and low-risk groups. **A** Erlotinib. **B** Gemcitabine. **C** Cytarabine. **D** Gefitinib. **E** MK-2206. **F** CCT007093

**Fig. 14:**
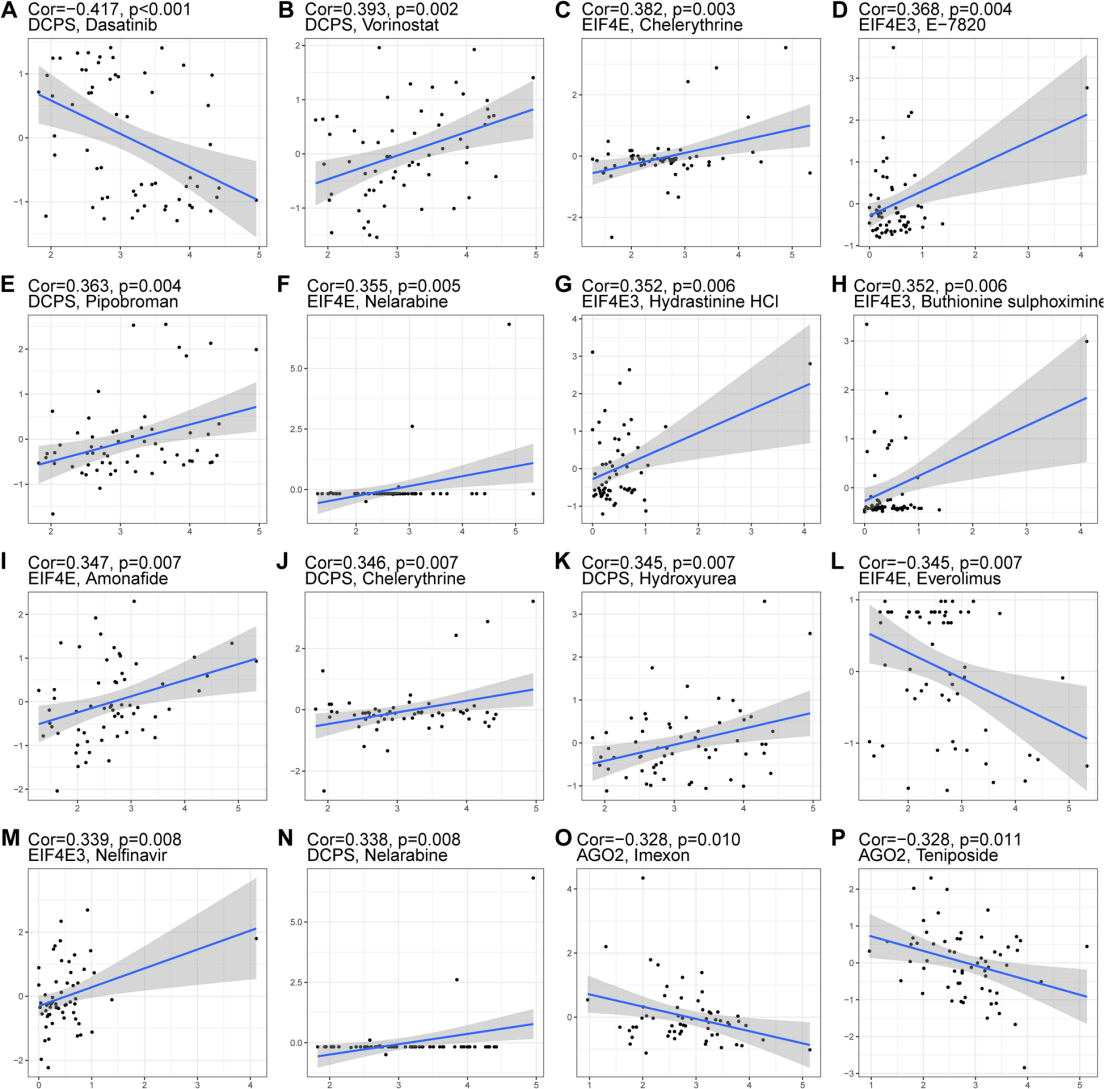
Correlations between the four prognostic m7GRGs and drug sensitivity in breast cancer. Correlations of DCPS with dasatinib (**A**), vorinostat (**B**), pipobroman (**E**), chelerythrine (**J**), hydroxyurea (**K**) and nelarabine (**N**). Correlations of EIF4E with chelerythrine (**C**), nelarabine (**F**), amonafide (**I**) and everolimus (**L**). Correlations of EIF4E3 with E-7820 (**D**), hydrastinine HCl (**G**), buthionine sulfoximine (**H**) and nelfinavir (**M**). Correlations of AGO2 with imexon (**O**) and teniposide (P)

To evaluate the drug prediction ability of the 4-m7GRG prognostic risk model, we used the ‘pRRophetic’ package to compare the differences in the estimated IC_50_ levels of six chemotherapeutic agents, namely, erlotinib (Fig. 13A), gemcitabine (Fig. 13B), cytarabine (Fig. 13C), gefitinib (Fig. 13D), the Akt1/2/3 inhibitor MK-2206 (Fig. 13E) and the PPM1D (WIP1) inhibitor CCT007093 (Fig. 13F). Our data showed that the high-risk score group was more sensitive to gemcitabine and cytarabine than the low-risk group. The above results indicated that the 4-m7GRG prognostic model has good drug sensitivity. Furthermore, to screen possible therapeutic drugs for breast cancer, based on the CellMiner database, we identified the drugs related to the risk score of the prognostic model. The results indicated that the low-risk score group was more sensitive to gefitinib and CCT007093. Drug sensitivity analysis of the four-gene signature showed that the DCPS, EIF4E, EIF4E3 and AGO2 genes were significantly associated with dasatinib, chelerythrine, E7820 and imexon, respectively (Fig. 14A–P).

### Expression of the signature m7GRGs

**Fig. 15:**
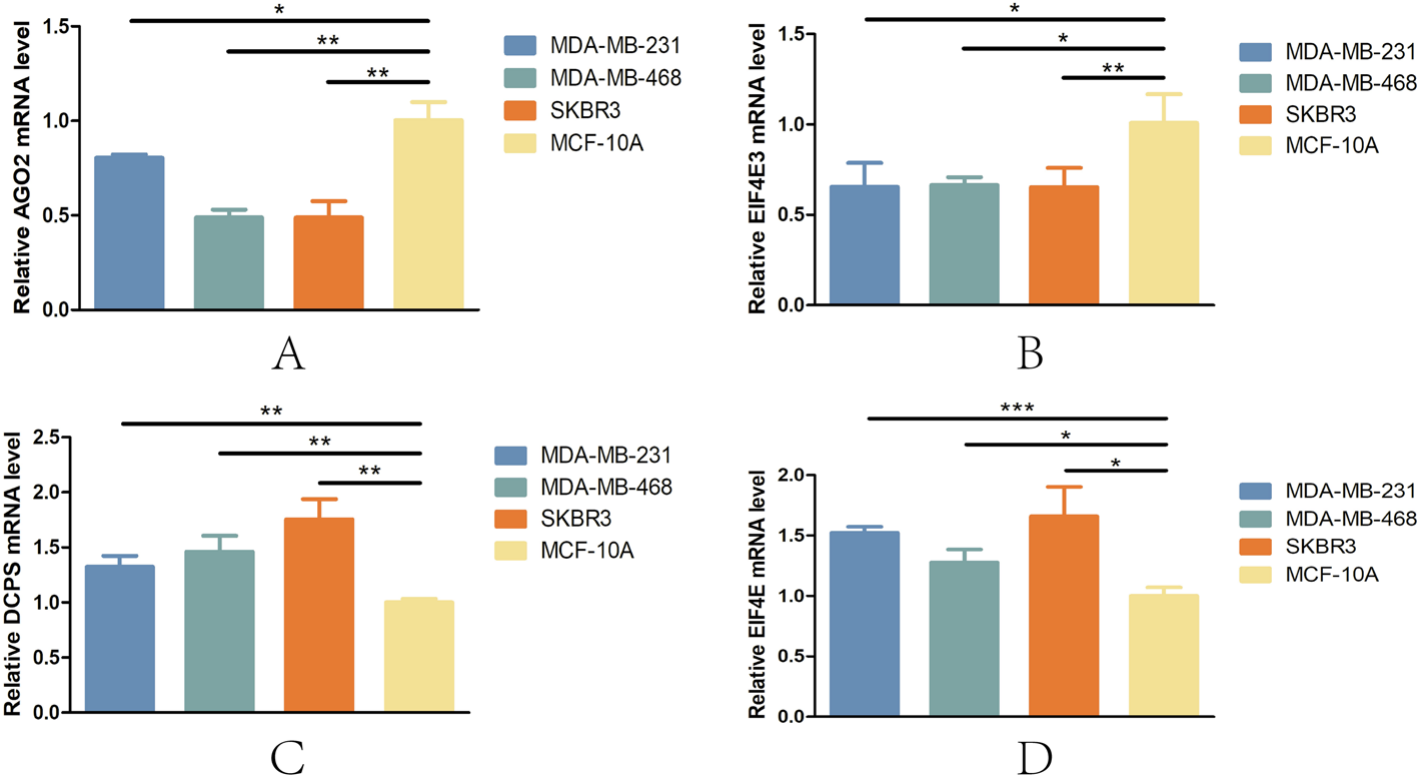
Expression of AGO2 (**A**), EIF4E3 (**B**), DCPS (**C**) and EIF4E (**D**) in breast cancer cell lines. *p < 0.05, **p < 0.01, ***p < 0.001

Breast cancer cell lines (MDA-MB-231, MDA-MB-468 and SKBR3) and a normal breast cell line (MCF-10A) were used to validate the expression levels of the signature m7GRGs (AGO2, EIF4E3, DCPS and EIF4E). The results showed that the expression levels of AGO2 (Fig. 15A) and EIF4E3 (Fig. 15B) were significantly lower in breast cancer cell lines than in the normal breast cell line, whereas the expression levels of DCPS (Fig. 15C) and EIF4E (Fig. 15D) were significantly higher. The above results were consistent with the results of the bioinformatics analysis. Therefore, further exploration of the exact mechanism of these four m7Gs in breast cancer would be of great significance for improving the prognosis and treatment of breast cancer patients. In addition, we also explored the expression of AGO2, EIF4E3, DCPS and EIF4E in pan-cancer (Supplementary Material S7).

## Discussion

Breast cancer is a solid tumour with high tumour heterogeneity, and effective treatments and prognostic biomarkers have not yet been found. Accordingly, the identification of innovative biomarkers is urgently required. m7G plays a major role in the occurrence, immune response and prognosis prediction of cancer and is a potential biomarker of cancer. Therefore, we aimed to clarify the role of m7G in breast cancer and identify biomarkers related to the disease.

First, to investigate the function of m7G modification in breast cancer, we identified the expression, CNV incidence and prognostic value of 24 m7GRGs in breast cancer. We found that the expression levels of NSUN2, EIF4E, EIF4E2, NCBP2, NUDT3, NCBP1, LARP1, WDR4 and LSM1 were increased in breast cancer tissues. Moreover, the results of prognostic analysis showed that more than half of the m7GRGs were risk factors for the prognosis of breast cancer patients. Therefore, it was deemed necessary to further explore the prognostic impact of m7GRGs in breast cancer.

Then, we performed consensus clustering and divided tumour samples into four subtypes based on the expression of the 24 m7GRGs in the TCGA-BRCA dataset. The expression of the 24 m7GRGs significantly differed among different breast cancer subtypes. The results of immune correlation analysis showed that there were significant differences in the infiltration level of immune cells and matrix score among the different subtypes of breast cancer. The immune cells exhibiting differences included B_cells_naïve, T_cells_CD8, T_cells_CD4_Naive, Macrophages (M0, M1 and M2), NK_cells_resting, NK_cells_Activated and other immune cells. Several studies of breast cancer have shown that B cell infiltration can predict a better survival rate and response to treatment [25]. Buque et al. found that NK cells have an inhibitory effect in the early stage of breast cancer [26]. Enrichment analysis results showed that the enrichment pathways also differed among different breast cancer subtypes, with some pathways related to immunity, such as T cell receptor complex, TNF signalling pathway and Salmonella infection. Most members of the TNF family and their receptors can affect the survival, proliferation, differentiation or activation of immune cells [27], and *Salmonella* can disrupt the host's immune defence during antibiotic treatment [28]. The above results showed that the 24 m7GRGs can appropriately identify different breast cancer subtypes and that patients belonging to the different cancer subtypes should undergo different treatment strategies.

Subsequently, based on the 24 m7GRGs, we screened 21 prognosis-related m7GRGs using the SVM algorithm. Multivariate Cox regression analysis was performed based on these 21 prognostic-related m7G genes, and four genes for constructing the prediction model were identified: AGO2, EIF4E3, DCPS and EIF4E. In our study, the samples were divided into high- and low-risk groups according to the median risk score, and survival analysis identified a significant survival difference between the two groups. Kaplan–Meier curve analysis revealed significant differences in OS between patients with high and low expression of AGO2, EIF4E3, DCPS and EIF4E. In addition, the DMFS rates of AGO2 and EIF4E3 were significantly different, which might be related to the metastasis of breast cancer. Multivariate Cox regression analysis showed that risk score, age and stage were independent predictors of breast cancer. Therefore, we constructed a nomogram based on risk score, age and stage. The calibration curve, ROC curve and C-index curve showed that the nomogram adequately predicted the prognosis of breast cancer patients. Overall, we have provided compelling evidence that the m7G gene signature is associated with the prognosis of breast cancer patients.

Our study found that the high-risk group had higher TMB levels than the low-risk group and that patients with high TMB levels had higher survival rates than those with low TMB levels. The above results suggest that high TMB may be associated with a poorer prognosis in high-risk patients. Moreover, the four-gene signature was able to predict the response to chemotherapy. Pearson analysis was used to calculate and obtain drugs (such as dasatinib, vorinostat and pipobroman) related to the risk score, which may become potential therapeutic drugs for breast cancer. In addition, PCR results showed that the expression levels of the four m7Gs were significantly different between breast cancer cell lines and normal breast cells (*p* < 0.05).

The m7GRGs AGO2, EIF4E3, DCPS and EIF4E have been extensively studied, and some of them have been associated with tumour progression. AGO2 is the only catalytically active member of the Argonaute family; it is involved in small RNA-guided post-transcriptional gene silencing (including mRNA degradation and translational repression) [29]. In addition, AGO2 plays multiple roles in nuclear gene regulation, such as chromatin remodelling, double-strand break repair and alternative splicing transcriptional repression and activation [30]. AGO2 may play a role in double-strand break repair in tumour cells. eIF4E is a eukaryotic translation initiation factor and an oncogene with elevated expression in approximately 30% of human cancers [31, 32]. Its elevation in mouse models is associated with tumorigenesis, and tissue culture experiments have shown that the expression of eIF4E is associated with oncogenic transformation. eIF4E functions in mRNA export and the translation of specific transcripts by binding to the methyl 7-guanosine cap found at the 5' end of mRNAs. These transcripts often encode proteins involved in proliferation, survival, invasion and metastasis. Unlike eIF4E1, eIF4E3 functions as a tissue-specific tumour suppressor [33]. In this respect, it has been shown that eIF4E3 inhibits the expression of both the mRNA export and translation targets of eIF4E1. The protein encoded by the DCPS gene is an mRNA decapping enzyme scavenger and is believed to be key for AML cell survival. Mass spectrometry analysis has revealed that DCPS enzymes interact with and function via components of the pre-mRNA metabolic pathway, including the spliceosome [34].

To conclude, we identified four subtypes of breast cancer based on 24 m7GRGs and found significant differences in the immune microenvironment and pathways among the different subtypes. This shows that different types of breast cancer patients need personalised treatment. Furthermore, we constructed a 4-m7GRG prognostic model that can predict the prognosis of breast cancer and clarified the differences in TMB level and drug sensitivity between risk subgroups. Importantly, our study provides a theoretical basis for applying the m7G gene signature for the personalised treatment of breast cancer patients and obtained novel insights into the mechanism underlying breast cancer progression and treatment.

## Supplementary Information

**Additional file 1: Supplementary Material S1.** The primers sequences (5’-3’). **Supplementary Material S2.** Expression of 24 m7GRGs in RNA-seq. **Supplementary Material S3.** The changes of DNA methylation in 18 m7GRGs. **Supplementary Material S4.** Cluster results of TCGA-BRCA queue. **Supplementary Material S5.** DMFS (Distant metastasis free survival) curve of AGO2, EIF4E3, and EIF4E. **Supplementary Material S6.** C index of nomogram. **Supplementary Material S7.** The expression landscapes of AGO2, EIF4E3, DCPS, and EIF4E in different cell lines based on the CCLE database.

## Data Availability

This paper involves all data come from the TCGA-BRCA queue of TCGA database (https://portal.gdc.cancer.gov/projects/TCGA-BRCA) and GEO database GSE1456 data set (https://www.ncbi.nlm.nih.gov/geo/query/acc.cgi?acc=GSE1456).

## References

[1] Ellsworth RE, Blackburn HL, Shriver CD, Soon-Shiong P, Ellsworth DL (2017). Molecular heterogeneity in breast cancer: State of the science and implications for patient care. Semin Cell Dev Biol.

[2] Litton JK, Burstein HJ, Turner NC (2019). Molecular Testing in Breast Cancer. Am Soc Clin Oncol Educ Book.

[3] Walker-Smith TL, Peck J (2019). Genetic and Genomic Advances in Breast Cancer Diagnosis and Treatment. Nurs Womens Health.

[4] Chlebowski RT, Manson JE (2022). Menopausal Hormone Therapy and Breast Cancer. Cancer J..

[5] Yamauchi H, Takei J (2018). Management of hereditary breast and ovarian cancer. Int J Clin Oncol.

[6] Cocco S, Leone A, Piezzo M, Caputo R, Di Lauro V, Di Rella F, Fusco G, Capozzi M, Gioia GD, Budillon A, De Laurentiis M (2020). Targeting Autophagy in Breast Cancer. Int J Mol Sci.

[7] Jiang YZ, Liu Y, Xiao Y, Hu X, Jiang L, Zuo WJ, Ma D, Ding J, Zhu X, Zou J, Verschraegen C, Stover DG, Kaklamani V, Wang ZH, Shao ZM (2021). Molecular subtyping and genomic profiling expand precision medicine in refractory metastatic triple-negative breast cancer: the FUTURE trial. Cell Res..

[8] Chen Y, Lin H, Miao L, He J (2022). Role of N7-methylguanosine (m7G) in cancer. Trends Cell Biol.

[9] Li J, Zhang H, Wang H (2022). N1-methyladenosine modification in cancer biology: Current status and future perspectives. Comput Struct Biotechnol J.

[10] Luo Y, Yao Y, Wu P, Zi X, Sun N, He J (2022). The potential role of N7-methylguanosine (m7G) in cancer. J Hematol Oncol.

[11] Wei W, Liu C, Wang C, Wang M, Jiang W, Zhou Y, Zhang S (2022). Comprehensive pan-cancer analysis of N7-methylguanosine regulators: Expression features and potential implications in prognosis and immunotherapy. Front Genet.

[12] Li XY, Wang SL, Chen DH, Liu H, You JX, Su LX, Yang XT (2022). Construction and Validation of a m7G-Related Gene-Based Prognostic Model for Gastric Cancer. Front Oncol..

[13] Du D, He J, Ju C, Wang C, Li H, He F, Zhou M (2023). When N7-methyladenosine modification meets cancer: Emerging frontiers and promising therapeutic opportunities. Cancer Lett..

[14] Halaby MJ, Hezaveh K, Lamorte S, Ciudad MT, Kloetgen A, MacLeod BL, Guo M, Chakravarthy A, Medina TDS, Ugel S, Tsirigos A, Bronte V, Munn DH, Pugh TJ, De Carvalho DD, Butler MO, Ohashi PS, Brooks DG, McGaha TL (2019). GCN2 drives macrophage and MDSC function and immunosuppression in the tumor microenvironment. Sci Immunol..

[15] Wilson JL, Nägele T, Linke M, Demel F, Fritsch SD, Mayr HK, Cai Z, Katholnig K, Sun X, Fragner L, Miller A, Haschemi A, Popa A, Bergthaler A, Hengstschläger M, Weichhart T, Weckwerth W (2020). Inverse Data-Driven Modeling and Multiomics Analysis Reveals Phgdh as a Metabolic Checkpoint of Macrophage Polarization and Proliferation. Cell Rep.

[16] Soysal SD, Tzankov A, Muenst SE (2015). Role of the Tumor Microenvironment in Breast Cancer. Pathobiology.

[17] Cui L, Ma R, Cai J, Guo C, Chen Z, Yao L, Wang Y, Fan R, Wang X, Shi Y (2022). RNA modifications: importance in immune cell biology and related diseases. Signal Transduct Target Ther.

[18] Zhang M, Song J, Yuan W, Zhang W, Sun Z (2021). Roles of RNA Methylation on Tumor Immunity and Clinical Implications. Front Immunol..

[19] Huang X, Chen Z, Xiang X, Liu Y, Long X, Li K, Qin M, Long C, Mo X, Tang W, Liu J (2022). Comprehensive multi-omics analysis of the m7G in pan-cancer from the perspective of predictive, preventive, and personalized medicine. EPMA J.

[20] Liu Y, Wang J, Li L, Qin H, Wei Y, Zhang X, Ren X, Ding W, Shen X, Li G, Lu Z, Zhang D, Qin C, Tao L, Chen X (2022). AC010973.2 promotes cell proliferation and is one of six stemness-related genes that predict overall survival of renal clear cell carcinoma. Sci Rep..

[21] Wu D, Yin Z, Ji Y, Li L, Li Y, Meng F, Ren X, Xu M (2021). Identification of novel autophagy-related lncRNAs associated with a poor prognosis of colon adenocarcinoma through bioinformatics analysis. Sci Rep.

[22] Tomikawa C (2018). 7-Methylguanosine Modifications in Transfer RNA (tRNA). Int J Mol Sci.

[23] Kanehisa M, Goto S (2000). KEGG: kyoto encyclopedia of genes and genomes. Nucleic Acids Res.

[24] Kanehisa M (2019). Toward understanding the origin and evolution of cellular organisms. Protein Sci..

[25] Zhang Z, Zhu Y, Wang Z, Zhang T, Wu P, Huang J (2017). Yin-yang effect of tumor infiltrating B cells in breast cancer: From mechanism to immunotherapy. Cancer Lett.

[26] Buque A, Bloy N, Petroni G, Kroemer G, Galluzzi L (2020). NK cells beat T cells at early breast cancer control. Oncoimmunology.

[27] Juhász K, Buzás K, Duda E (2013). Importance of reverse signaling of the TNF superfamily in immune regulation. Expert Rev Clin Immunol.

[28] Stapels DAC, Hill PWS, Westermann AJ, Fisher RA, Thurston TL, Saliba AE, Blommestein I, Vogel J, Helaine S (2018). Salmonella persisters undermine host immune defenses during antibiotic treatment. Science.

[29] Li X, Wang X, Cheng Z, Zhu Q (2020). AGO2 and its partners: a silencing complex, a chromatin modulator, and new features. Crit Rev Biochem Mol Biol.

[30] Müller M, Fäh T, Schaefer M, Hermes V, Luitz J, Stalder P, Arora R, Ngondo RP, Ciaudo C (2022). AGO1 regulates pericentromeric regions in mouse embryonic stem cells. Life Sci Alliance..

[31] Graff JR, Zimmer SG (2003). Translational control and metastatic progression: enhanced activity of the mRNA cap-binding protein eIF-4E selectively enhances translation of metastasis-related mRNAs. Clin Exp Metastasis.

[32] Topisirovic I, Guzman ML, McConnell MJ, Licht JD, Culjkovic B, Neering SJ, Jordan CT, Borden KL (2003). Aberrant eukaryotic translation initiation factor 4E-dependent mRNA transport impedes hematopoietic differentiation and contributes to leukemogenesis. Mol Cell Biol.

[33] Osborne MJ, Volpon L, Kornblatt JA, Culjkovic-Kraljacic B, Baguet A, Borden KL (2013). eIF4E3 acts as a tumor suppressor by utilizing an atypical mode of methyl-7-guanosine cap recognition. Proc Natl Acad Sci U S A..

[34] Yamauchi T, Masuda T, Canver MC, Seiler M, Semba Y, Shboul M, Al-Raqad M, Maeda M, Schoonenberg VAC, Cole MA, Macias-Trevino C, Ishikawa Y, Yao Q, Nakano M, Arai F, Orkin SH, Reversade B, Buonamici S, Pinello L, Akashi K, Bauer DE, Maeda T (2018). Genome-wide CRISPR-Cas9 Screen Identifies Leukemia-Specific Dependence on a Pre-mRNA Metabolic Pathway Regulated by DCPS. Cancer Cell..

