# Supplementary material for: Analysis of m7G-Related signatures in the tumour immune microenvironment and identification of clinical prognostic regulators in breast cancer
Source: BMC Cancer. 2023 Jun 23;23:583. doi: 10.1186/s12885-023-11012-z (PMC10288749; doi:10.1186/s12885-023-11012-z)
Supplement: Supplementary file 1 — Additional file 1: Supplementary Material S1. The primers sequences (5’-3’). Supplementary Material S2. Expression of 24 m7GRGs in RNA-seq. Supplementary Material S3. The changes of DNA methylation in 18 m7GRGs. Supplementary Material S4. Cluster results of TCGA-BRCA queue. Supplementary Material S5. DMFS (Distant metastasis free survival) curve of AGO2, EIF4E3, and EIF4E. Supplementary Material S6. C index of nomogram. Supplementary Material S7. The expression landscapes of AGO2, EIF4E3, DCPS, and EIF4E in different cell lines based on the CCLE database. [file 12885_2023_11012_MOESM1_ESM.pdf]

Supplementary Material S1: The primers sequences (5’-3’)

AGO2

**Forward primer**	TCCACCTAGACCCGACTTTGG
**Reverse primer**	GTGTTCCACGATTTCCCTGTT

EIF4E3

**Forward primer**	GCAAAGGGTGGCGTATGGAA
**Reverse primer**	CCCGATGGTTGCTAACAGC

EIF4E

**Forward primer**	GAAACCACCCCTACTCCTAATCC
**Reverse primer**	AGAGTGCCCATCTGTTCTGTA

DCPS

**Forward primer**	GCAGCTCCTCAACTAGGCAAG
**Reverse primer**	GAAGCCGGAGAACGGTAAGC

Supplementary Material S2：Expression of 24 m7GRGs in RNA-seq.

gene	conMean	treatMean	logFC	pValue	fdr
DCP2	2.497743	2.548924	0.029263	0.430586	0.449307
IFIT5	3.068548	3.122906	0.025333	0.711143	0.711143
EIF3D	6.184146	5.808371	-0.09044	3.76E-13	7.51E-13
EIF4G3	3.400347	3.458298	0.02438	0.10058	0.120696
NSUN2	3.613179	4.002495	0.147631	1.03E-17	2.25E-17
GEMIN5	3.040677	2.931432	-0.05279	0.005423	0.007231
AGO2	2.380648	2.373953	-0.00406	0.109148	0.124741
NUDT10	0.902379	0.785625	-0.19989	2.10E-06	3.36E-06
EIF4E	1.719527	1.916329	0.156333	1.89E-09	3.24E-09
EIF4E2	3.160464	3.437258	0.121122	8.78E-25	5.27E-24
NCBP2	4.077928	4.472261	0.133168	9.58E-27	7.66E-26
NUDT11	1.209852	0.818136	-0.56442	7.16E-22	2.45E-21
NUDT3	0.056035	0.109145	0.961855	2.22E-18	5.33E-18
NCBP1	3.189531	3.569618	0.162425	1.39E-20	3.70E-20
METTL1	2.602957	3.241659	0.316581	7.95E-30	9.54E-29
LARP1	4.64933	4.897966	0.07516	6.24E-11	1.15E-10
NUDT4	2.649405	2.547042	-0.05685	0.022936	0.028972
EIF4E3	2.854685	2.022631	-0.4971	1.44E-44	3.45E-43
SNUPN	2.011552	1.984982	-0.01918	0.241631	0.263597
WDR4	1.852233	2.375233	0.358803	9.94E-22	2.98E-21
LSM1	2.515897	3.105072	0.303554	1.98E-24	9.50E-24
NUDT16	3.407635	2.991726	-0.18779	1.09E-23	4.35E-23
DCPS	1.884296	2.048251	0.120367	0.00024	0.000338
CYFIP1	4.316232	4.167891	-0.05046	1.57E-05	2.35E-05

Supplementary Material S3: The changes of DNA methylation in 18 m7GRGs

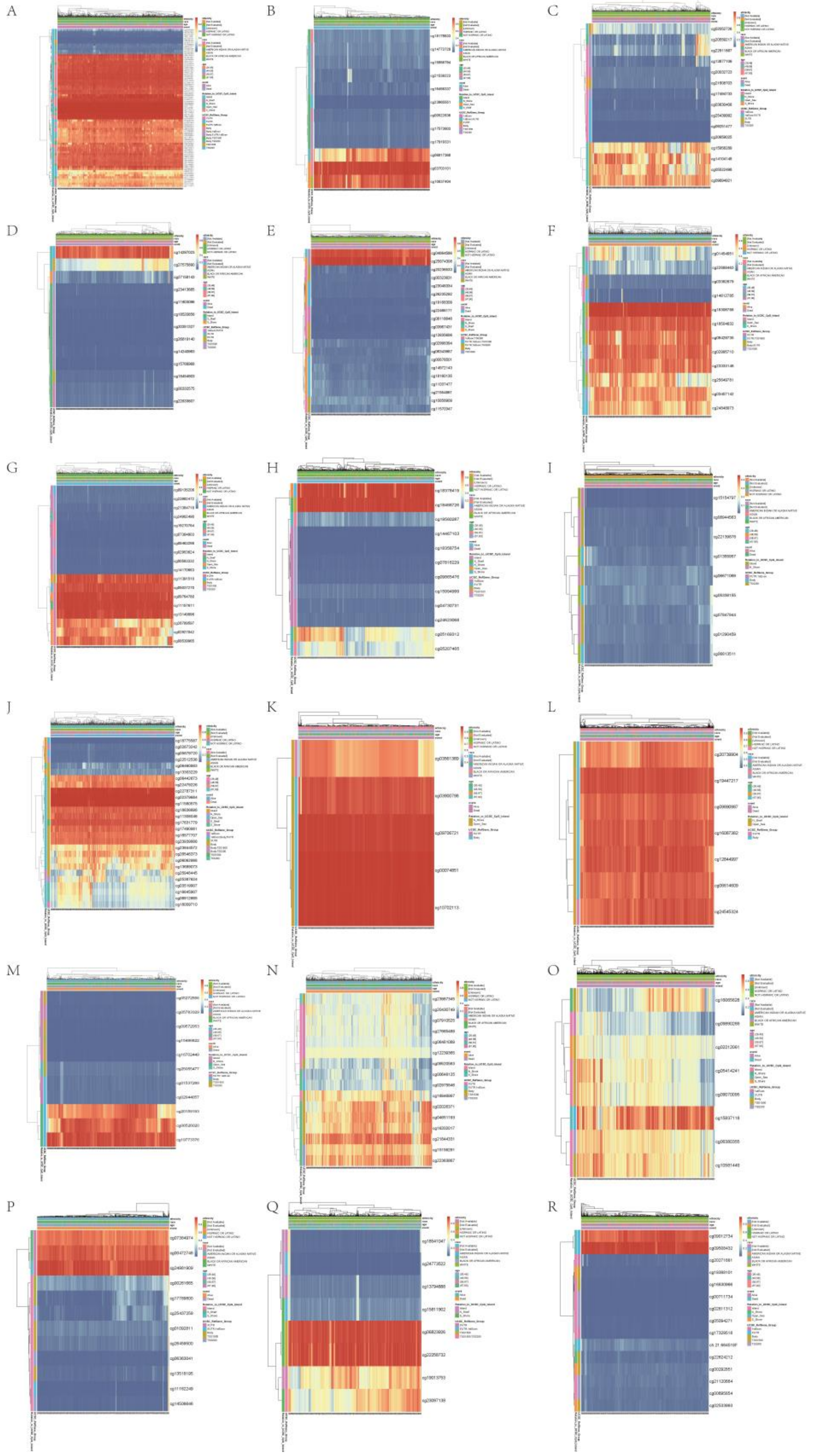

Figure 1 The changes of DNA methylation in 18 m7GRGs：CYFIP1(A), DCP2 (B), DCPS (C), EIF3D (D), EIF4E (E), EIF4E3 (F), EIF4G3 (G), GEMIN5 (H), IFIT5 (I), LARP1 (J), NCBP2 (K), NSUN2 (L), NUDT3 (M), NUDT10 (N), NUDT11 (O), NUDT16 (P), SNUPN (Q), WDR4 (R).

Supplementary Material S4: Cluster results of TCGA-BRCA queue.

sample	Subtype
TCGA-A8-A08A-01A	C1
TCGA-A8-A0A7-01A	C1
TCGA-A8-A09W-01A	C1
TCGA-A8-A08L-01A	C1
TCGA-E9-A1RD-01A	C1
TCGA-AC-A8OR-01A	C1
TCGA-AN-A04C-01A	C1
TCGA-E9-A248-01A	C1
TCGA-BH-A0E0-01A	C1
TCGA-AR-A0TR-01A	C1
TCGA-AN-A0XL-01A	C1
TCGA-BH-A0RX-01A	C1
TCGA-AN-A0XW-01A	C1
TCGA-AC-A3EH-01A	C1
TCGA-E9-A249-01A	C1
TCGA-AN-A0AK-01A	C1
TCGA-AN-A0FL-01A	C1
TCGA-AN-A0FJ-01A	C1
TCGA-A8-A07S-01A	C1
TCGA-E2-A1LK-01A	C1
TCGA-A8-A07R-01A	C1
TCGA-E2-A15O-01A	C1
TCGA-BH-A0E6-01A	C1
TCGA-C8-A278-01A	C1
TCGA-D8-A27R-01A	C1
TCGA-A7-A6VX-01A	C1
TCGA-BH-A0GZ-01A	C1
TCGA-A7-A5ZX-01A	C1
TCGA-A7-A3J1-01A	C1
TCGA-C8-A12M-01A	C1
TCGA-C8-A12P-01A	C1
TCGA-JL-A3YW-01A	C1
TCGA-B6-A0I9-01A	C1
TCGA-A7-A5ZV-01A	C1
TCGA-C8-A130-01A	C1
TCGA-EW-A1IW-01A	C1
TCGA-C8-A1HM-01A	C1
TCGA-C8-A138-01A	C1
TCGA-D8-A27V-01A	C1
TCGA-C8-A12V-01A	C1
TCGA-C8-A12O-01A	C1

TCGA-E2-A15H-01A	C1
TCGA-C8-A135-01A	C1
TCGA-C8-A1HN-01A	C1
TCGA-D8-A27H-01A	C1
TCGA-D8-A1JT-01A	C1
TCGA-A7-A3RF-01A	C1
TCGA-C8-A8HR-01A	C1
TCGA-D8-A27G-01A	C1
TCGA-D8-A145-01A	C1
TCGA-AC-A6IW-01A	C1
TCGA-BH-A0HP-01A	C1
TCGA-AC-A62X-01A	C1
TCGA-S3-AA11-01A	C1
TCGA-BH-A0DK-01A	C1
TCGA-D8-A142-01A	C1
TCGA-D8-A1JH-01A	C1
TCGA-E2-A15S-01A	C1
TCGA-OL-A66P-01A	C1
TCGA-D8-A1Y3-01A	C1
TCGA-BH-A0E2-01A	C1
TCGA-A7-A4SD-01A	C1
TCGA-AC-A2QJ-01A	C1
TCGA-E2-A158-01A	C1
TCGA-AC-A3OD-01B	C1
TCGA-A8-A09V-01A	C1
TCGA-BH-A0H0-01A	C1
TCGA-EW-A6S9-01A	C1
TCGA-BH-A18N-01A	C1
TCGA-AC-A3W7-01A	C1
TCGA-BH-A0E1-01A	C1
TCGA-XX-A89A-01A	C1
TCGA-BH-A0DZ-01A	C1
TCGA-D8-A1XS-01A	C1
TCGA-AC-A3HN-01A	C1
TCGA-D8-A27L-01A	C1
TCGA-D8-A1XY-01A	C1
TCGA-AC-A3W5-01A	C1
TCGA-BH-A0HN-01A	C1
TCGA-A2-A0T1-01A	C1
TCGA-A2-A1FW-01A	C1
TCGA-S3-AA14-01A	C1
TCGA-D8-A27E-01A	C1
TCGA-A2-A0T5-01A	C1

TCGA-EW-A423-01A	C1
TCGA-A8-A082-01A	C1
TCGA-D8-A1XB-01A	C1
TCGA-EW-A1P6-01A	C1
TCGA-S3-A6ZG-01A	C1
TCGA-AC-A6IV-01A	C1
TCGA-B6-A0IK-01A	C1
TCGA-BH-A8FZ-01A	C1
TCGA-EW-A1PA-01A	C1
TCGA-BH-A1FG-01A	C1
TCGA-A2-A1G1-01A	C1
TCGA-S3-AA10-01A	C1
TCGA-AC-A5XS-01A	C1
TCGA-D8-A1JM-01A	C1
TCGA-A2-A1G4-01A	C1
TCGA-LL-A8F5-01A	C1
TCGA-A8-A08G-01A	C1
TCGA-BH-A2L8-01A	C1
TCGA-A7-A13E-01B	C1
TCGA-A2-A1G0-01A	C1
TCGA-AR-A2LH-01A	C1
TCGA-D8-A1JN-01A	C1
TCGA-OL-A5S0-01A	C1
TCGA-E2-A15L-01A	C1
TCGA-A7-A26J-01A	C1
TCGA-E2-A15E-01A	C1
TCGA-A2-A0T7-01A	C1
TCGA-BH-A42V-01A	C1
TCGA-A2-A4S2-01A	C1
TCGA-D8-A146-01A	C1
TCGA-BH-A18H-01A	C1
TCGA-E2-A15F-01A	C1
TCGA-D8-A4Z1-01A	C1
TCGA-BH-A0BJ-01A	C1
TCGA-A2-A4S3-01A	C1
TCGA-A2-A1FZ-01A	C1
TCGA-BH-A0WA-01A	C1
TCGA-BH-A0H7-01A	C1
TCGA-A2-A4S0-01A	C1
TCGA-OL-A66I-01A	C1
TCGA-A2-A1FV-01A	C1
TCGA-BH-A5J0-01A	C1
TCGA-A7-A13G-01B	C1

TCGA-A7-A26G-01A	C1
TCGA-E9-A54Y-01A	C1
TCGA-BH-A0EB-01A	C1
TCGA-BH-A0H6-01A	C1
TCGA-AC-A3YJ-01A	C1
TCGA-E9-A1QZ-01A	C1
TCGA-EW-A6SB-01A	C1
TCGA-A7-A13F-01A	C1
TCGA-D8-A73X-01A	C1
TCGA-A2-A0D4-01A	C1
TCGA-D8-A1X8-01A	C1
TCGA-BH-A1F0-01A	C1
TCGA-E9-A1NG-01A	C1
TCGA-A8-A06Y-01A	C1
TCGA-BH-A202-01A	C1
TCGA-E9-A5UP-01A	C1
TCGA-OL-A66H-01A	C1
TCGA-AQ-A04J-01A	C1
TCGA-D8-A1XA-01A	C1
TCGA-BH-A201-01A	C1
TCGA-B6-A0X4-01A	C1
TCGA-A7-A0DC-01A	C1
TCGA-A7-A0DC-01B	C1
TCGA-EW-A1P4-01A	C1
TCGA-EW-A1OX-01A	C1
TCGA-BH-A0DI-01A	C1
TCGA-A8-A09K-01A	C1
TCGA-A2-A3XU-01A	C1
TCGA-BH-A18P-01A	C1
TCGA-E2-A570-01A	C1
TCGA-A8-A08P-01A	C1
TCGA-A8-A06X-01A	C1
TCGA-A7-A26E-01A	C1
TCGA-BH-A1F2-01A	C1
TCGA-A2-A0EV-01A	C1
TCGA-BH-A0BC-01A	C1
TCGA-AQ-A1H3-01A	C1
TCGA-A2-A0YC-01A	C1
TCGA-B6-A0I6-01A	C1
TCGA-A2-A3XV-01A	C1
TCGA-LL-A441-01A	C1
TCGA-AO-A0J2-01A	C1
TCGA-BH-A18F-01A	C1

TCGA-AQ-A54O-01A	C1
TCGA-A8-A08F-01A	C1
TCGA-E2-A1B4-01A	C1
TCGA-AC-A2QH-01B	C1
TCGA-A7-A0DB-01C	C1
TCGA-EW-A6SD-01A	C1
TCGA-E9-A1N8-01A	C1
TCGA-A2-A0EU-01A	C1
TCGA-E9-A1NF-01A	C1
TCGA-BH-A18I-01A	C1
TCGA-E9-A1N5-01A	C1
TCGA-BH-A28O-01A	C1
TCGA-A8-A08J-01A	C1
TCGA-AO-A0J6-01A	C1
TCGA-E2-A10B-01A	C1
TCGA-GM-A3NY-01A	C1
TCGA-A7-A0CD-01A	C1
TCGA-E9-A22B-01A	C1
TCGA-E9-A22A-01A	C1
TCGA-A1-A0SN-01A	C1
TCGA-BH-A0B3-01A	C1
TCGA-A2-A3KD-01A	C1
TCGA-EW-A1P1-01A	C1
TCGA-EW-A1OZ-01A	C1
TCGA-AO-A12H-01A	C1
TCGA-EW-A1P0-01A	C1
TCGA-E9-A1ND-01A	C1
TCGA-AR-A1AT-01A	C1
TCGA-A8-A095-01A	C1
TCGA-E9-A228-01A	C1
TCGA-E2-A1LL-01A	C1
TCGA-BH-A0BF-01A	C1
TCGA-GM-A3XG-01A	C1
TCGA-AO-A03V-01A	C1
TCGA-E2-A14O-01A	C1
TCGA-BH-A0E7-01A	C1
TCGA-E9-A1RA-01A	C1
TCGA-BH-A1FM-01A	C1
TCGA-E9-A1RH-01A	C1
TCGA-E9-A1RE-01A	C1
TCGA-A1-A0SH-01A	C1
TCGA-A1-A0SF-01A	C1
TCGA-A2-A0CO-01A	C1

TCGA-5L-AAT1-01A	C1
TCGA-3C-AALJ-01A	C1
TCGA-BH-A1EX-01A	C1
TCGA-AO-A0JI-01A	C1
TCGA-A2-A3XZ-01A	C1
TCGA-AO-A0JB-01A	C1
TCGA-AO-A0JC-01A	C1
TCGA-A2-A259-01A	C1
TCGA-D8-A1JG-01B	C1
TCGA-AO-A0J9-01A	C1
TCGA-E2-A1L6-01A	C1
TCGA-EW-A2FR-01A	C1
TCGA-A2-A0CY-01A	C1
TCGA-D8-A1XO-01A	C1
TCGA-BH-A1FU-01A	C1
TCGA-E2-A15I-01A	C1
TCGA-A2-A3XW-01A	C1
TCGA-AR-A2LR-01A	C1
TCGA-BH-A208-01A	C1
TCGA-OL-A5D7-01A	C1
TCGA-OL-A5DA-01A	C1
TCGA-GM-A2D9-01A	C1
TCGA-E2-A1L7-01A	C1
TCGA-AR-A1AV-01A	C1
TCGA-HN-A2OB-01A	C1
TCGA-BH-A0AZ-01A	C1
TCGA-A2-A0EY-01A	C1
TCGA-AR-A0U0-01A	C1
TCGA-OL-A66J-01A	C1
TCGA-V7-A7HQ-01A	C1
TCGA-A2-A0D0-01A	C1
TCGA-B6-A408-01A	C1
TCGA-B6-A0RG-01A	C1
TCGA-AO-A03R-01A	C1
TCGA-BH-A0B5-01A	C1
TCGA-AO-A0JM-01A	C1
TCGA-A2-A0ES-01A	C1
TCGA-GM-A4E0-01A	C1
TCGA-BH-A1FN-01A	C1
TCGA-AC-A2BK-01A	C1
TCGA-E2-A1L8-01A	C1
TCGA-BH-A0BZ-01A	C1
TCGA-A2-A0ER-01A	C1

TCGA-B6-A402-01A	C1
TCGA-GM-A2DD-01A	C1
TCGA-A2-A0CT-01A	C1
TCGA-E2-A1IE-01A	C1
TCGA-BH-A0BW-01A	C1
TCGA-BH-A0DE-01A	C1
TCGA-AR-A1AJ-01A	C1
TCGA-AO-A03L-01A	C1
TCGA-B6-A0RL-01A	C1
TCGA-BH-A0DD-01A	C1
TCGA-AO-A1KR-01A	C1
TCGA-BH-A1ET-01A	C1
TCGA-AR-A0TS-01A	C1
TCGA-B6-A401-01A	C1
TCGA-AR-A254-01A	C1
TCGA-A2-A04U-01A	C1
TCGA-A2-A0CQ-01A	C1
TCGA-BH-A0C3-01A	C1
TCGA-BH-A0C7-01B	C1
TCGA-A2-A3XT-01A	C1
TCGA-A2-A0CP-01A	C1
TCGA-B6-A0IH-01A	C1
TCGA-AR-A1AM-01A	C1
TCGA-AR-A24Z-01A	C1
TCGA-AR-A24X-01A	C1
TCGA-AR-A0TW-01A	C1
TCGA-A2-A0CL-01A	C1
TCGA-AO-A12A-01A	C1
TCGA-E2-A1LI-01A	C1
TCGA-GM-A2DM-01A	C1
TCGA-E2-A1LH-01A	C1
TCGA-AO-A129-01A	C1
TCGA-AR-A0TT-01A	C1
TCGA-GM-A3NW-01A	C1
TCGA-BH-A42U-01A	C1
TCGA-AO-A125-01A	C1
TCGA-BH-A1FC-01A	C1
TCGA-AO-A124-01A	C1
TCGA-B6-A0IM-01A	C1
TCGA-B6-A0X0-01A	C1
TCGA-B6-A1KN-01A	C1
TCGA-B6-A0RQ-01A	C1
TCGA-A2-A04N-01A	C1

TCGA-B6-A0IG-01A	C1
TCGA-AR-A2LE-01A	C1
TCGA-GM-A2DA-01A	C1
TCGA-B6-A0RN-01A	C1
TCGA-A8-A08R-01A	C2
TCGA-A8-A09N-01A	C2
TCGA-AC-A8OQ-01A	C2
TCGA-D8-A27P-01A	C2
TCGA-AC-A6NO-01A	C2
TCGA-AQ-A54N-01A	C2
TCGA-HN-A2NL-01A	C2
TCGA-AN-A04A-01A	C2
TCGA-E2-A1IL-01A	C2
TCGA-BH-A0HK-01A	C2
TCGA-EW-A1PC-01B	C2
TCGA-AN-A0FS-01A	C2
TCGA-AN-A0FK-01A	C2
TCGA-AN-A0FT-01A	C2
TCGA-B6-A400-01A	C2
TCGA-GI-A2C8-01A	C2
TCGA-AN-A0AL-01A	C2
TCGA-LL-A73Z-01A	C2
TCGA-A2-A0T2-01A	C2
TCGA-AN-A0AJ-01A	C2
TCGA-A8-A07O-01A	C2
TCGA-A8-A07W-01A	C2
TCGA-A7-A6VV-01A	C2
TCGA-A7-A3J0-01A	C2
TCGA-C8-A1HL-01A	C2
TCGA-EW-A1PE-01A	C2
TCGA-BH-A42T-01A	C2
TCGA-AR-A5QQ-01A	C2
TCGA-A2-A25F-01A	C2
TCGA-A7-A3IZ-01A	C2
TCGA-A7-A5ZW-01A	C2
TCGA-E2-A15M-01A	C2
TCGA-E9-A1R6-01A	C2
TCGA-C8-A1HG-01A	C2
TCGA-W8-A86G-01A	C2
TCGA-4H-AAAK-01A	C2
TCGA-AO-A1KS-01A	C2
TCGA-C8-A12N-01A	C2
TCGA-C8-A12L-01A	C2

TCGA-A8-A09B-01A	C2
TCGA-D8-A1JS-01A	C2
TCGA-C8-A26X-01A	C2
TCGA-LL-A7T0-01A	C2
TCGA-D8-A1XC-01A	C2
TCGA-C8-A8HQ-01A	C2
TCGA-BH-A0HU-01A	C2
TCGA-A8-A086-01A	C2
TCGA-LD-A74U-01A	C2
TCGA-C8-A131-01A	C2
TCGA-A7-A4SB-01A	C2
TCGA-EW-A1PD-01A	C2
TCGA-A8-A09X-01A	C2
TCGA-D8-A143-01A	C2
TCGA-D8-A1J8-01A	C2
TCGA-A1-A0SG-01A	C2
TCGA-EW-A1PF-01A	C2
TCGA-LL-A740-01A	C2
TCGA-D8-A1XK-01A	C2
TCGA-A7-A425-01A	C2
TCGA-D8-A1JU-01A	C2
TCGA-A8-A084-01A	C2
TCGA-XX-A899-01A	C2
TCGA-C8-A26Z-01A	C2
TCGA-EW-A1J5-01A	C2
TCGA-LL-A73Y-01A	C2
TCGA-D8-A1JC-01A	C2
TCGA-D8-A1XR-01A	C2
TCGA-D8-A1XQ-01A	C2
TCGA-EW-A1J3-01A	C2
TCGA-EW-A6SA-01A	C2
TCGA-A8-A0AB-01A	C2
TCGA-LL-A5YL-01A	C2
TCGA-D8-A1XD-01A	C2
TCGA-EW-A3U0-01A	C2
TCGA-D8-A1J9-01A	C2
TCGA-A7-A4SF-01A	C2
TCGA-A8-A06R-01A	C2
TCGA-A2-A04P-01A	C2
TCGA-D8-A1JD-01A	C2
TCGA-E2-A15G-01A	C2
TCGA-A1-A0SQ-01A	C2
TCGA-D8-A3Z6-01A	C2

TCGA-A2-A0YJ-01A	C2
TCGA-S3-A6ZF-01A	C2
TCGA-B6-A409-01A	C2
TCGA-D8-A1JE-01A	C2
TCGA-EW-A1J1-01A	C2
TCGA-A8-A07F-01A	C2
TCGA-D8-A1JI-01A	C2
TCGA-A1-A0SP-01A	C2
TCGA-A2-A0YK-01A	C2
TCGA-E2-A154-01A	C2
TCGA-OL-A6VQ-01A	C2
TCGA-D8-A1XL-01A	C2
TCGA-EW-A1PB-01A	C2
TCGA-D8-A1JL-01A	C2
TCGA-D8-A1JJ-01A	C2
TCGA-BH-A18J-01A	C2
TCGA-AC-A8OP-01A	C2
TCGA-AO-A1KO-01A	C2
TCGA-A2-A0T4-01A	C2
TCGA-A7-A26J-01B	C2
TCGA-A1-A0SI-01A	C2
TCGA-D8-A1JP-01A	C2
TCGA-B6-A0WX-01A	C2
TCGA-S3-A6ZH-01A	C2
TCGA-A7-A4SE-01A	C2
TCGA-LD-A66U-01A	C2
TCGA-E9-A1RG-01A	C2
TCGA-A2-A4RY-01A	C2
TCGA-A2-A0YH-01A	C2
TCGA-BH-A8G0-01A	C2
TCGA-A2-A0YG-01A	C2
TCGA-EW-A2FW-01A	C2
TCGA-AC-A2B8-01A	C2
TCGA-A7-A2KD-01A	C2
TCGA-AO-A0J8-01A	C2
TCGA-EW-A1OW-01A	C2
TCGA-E2-A153-01A	C2
TCGA-A2-A0YT-01A	C2
TCGA-A7-A26H-01A	C2
TCGA-A2-A4RX-01A	C2
TCGA-C8-A27A-01A	C2
TCGA-AQ-A04H-01B	C2
TCGA-BH-A0W4-01A	C2

TCGA-A8-A07U-01A	C2
TCGA-LL-A50Y-01A	C2
TCGA-E2-A159-01A	C2
TCGA-EW-A2FV-01A	C2
TCGA-EW-A1OV-01A	C2
TCGA-AC-A2FM-01A	C2
TCGA-OL-A66N-01A	C2
TCGA-AO-A0J5-01A	C2
TCGA-E9-A5FK-01A	C2
TCGA-A2-A4S1-01A	C2
TCGA-BH-A0HX-01A	C2
TCGA-E9-A6HE-01A	C2
TCGA-LQ-A4E4-01A	C2
TCGA-E2-A1IJ-01A	C2
TCGA-EW-A1J6-01A	C2
TCGA-E2-A10F-01A	C2
TCGA-E2-A1LE-01A	C2
TCGA-A8-A06U-01A	C2
TCGA-LL-A442-01A	C2
TCGA-A7-A13H-01A	C2
TCGA-E9-A3X8-01A	C2
TCGA-A8-A092-01A	C2
TCGA-A7-A13D-01B	C2
TCGA-A7-A13D-01A	C2
TCGA-A1-A0SK-01A	C2
TCGA-E9-A1RB-01A	C2
TCGA-AC-A3BB-01A	C2
TCGA-E9-A1N4-01A	C2
TCGA-A8-A091-01A	C2
TCGA-A8-A07C-01A	C2
TCGA-EW-A3E8-01B	C2
TCGA-E2-A576-01A	C2
TCGA-A7-A0CG-01A	C2
TCGA-E2-A107-01A	C2
TCGA-E9-A226-01A	C2
TCGA-EW-A1PG-01A	C2
TCGA-E9-A1N3-01A	C2
TCGA-A7-A0CE-01A	C2
TCGA-A7-A0CH-01A	C2
TCGA-A2-A3XY-01A	C2
TCGA-A2-A04Y-01A	C2
TCGA-E9-A1N9-01A	C2
TCGA-A2-A3KC-01A	C2

TCGA-E9-A1NA-01A	C2
TCGA-A8-A085-01A	C2
TCGA-E2-A1BD-01A	C2
TCGA-E2-A1IF-01A	C2
TCGA-E9-A229-01A	C2
TCGA-BH-A0B1-01A	C2
TCGA-AR-A1AS-01A	C2
TCGA-B6-A3ZX-01A	C2
TCGA-E9-A3HO-01A	C2
TCGA-E2-A14Q-01A	C2
TCGA-E2-A574-01A	C2
TCGA-AR-A5QP-01A	C2
TCGA-EW-A1IX-01A	C2
TCGA-E2-A10C-01A	C2
TCGA-BH-A0H9-01A	C2
TCGA-OL-A66K-01A	C2
TCGA-BH-A0W5-01A	C2
TCGA-OL-A66L-01A	C2
TCGA-A1-A0SE-01A	C2
TCGA-A2-A0SY-01A	C2
TCGA-BH-A0W7-01A	C2
TCGA-A2-A0SW-01A	C2
TCGA-A8-A07Z-01A	C2
TCGA-PE-A5DC-01A	C2
TCGA-3C-AALK-01A	C2
TCGA-A2-A0YL-01A	C2
TCGA-5L-AAT0-01A	C2
TCGA-A2-A0YF-01A	C2
TCGA-BH-A0HY-01A	C2
TCGA-BH-A18V-01A	C2
TCGA-E2-A1LS-01A	C2
TCGA-WT-AB41-01A	C2
TCGA-A2-A0CZ-01A	C2
TCGA-BH-A0H5-01A	C2
TCGA-E2-A15J-01A	C2
TCGA-BH-A1FR-01A	C2
TCGA-BH-A0DO-01B	C2
TCGA-AO-A0JL-01A	C2
TCGA-A2-A04X-01A	C2
TCGA-E2-A1IK-01A	C2
TCGA-A2-A1FX-01A	C2
TCGA-E2-A1IO-01A	C2
TCGA-AO-A03M-01B	C2

TCGA-BH-A0BG-01A	C2
TCGA-AO-A1KQ-01A	C2
TCGA-AO-A0JJ-01A	C2
TCGA-BH-A0EI-01A	C2
TCGA-BH-A0H3-01A	C2
TCGA-E2-A150-01A	C2
TCGA-BH-A18S-01A	C2
TCGA-AR-A2LL-01A	C2
TCGA-GM-A3XN-01A	C2
TCGA-B6-A0X5-01A	C2
TCGA-GM-A3XL-01A	C2
TCGA-BH-A0DX-01A	C2
TCGA-B6-A40C-01A	C2
TCGA-AO-A0JD-01A	C2
TCGA-GM-A2DH-01A	C2
TCGA-BH-A0BO-01A	C2
TCGA-AR-A5QM-01A	C2
TCGA-A2-A04T-01A	C2
TCGA-BH-A0BQ-01A	C2
TCGA-BH-A0BP-01A	C2
TCGA-A2-A0CS-01A	C2
TCGA-B6-A0I1-01A	C2
TCGA-BH-A0BT-01A	C2
TCGA-AO-A12C-01A	C2
TCGA-B6-A0WV-01A	C2
TCGA-A2-A0EO-01A	C2
TCGA-AO-A03O-01A	C2
TCGA-AO-A12D-01A	C2
TCGA-BH-A204-01A	C2
TCGA-E2-A106-01A	C2
TCGA-AR-A0U2-01A	C2
TCGA-GM-A2DO-01A	C2
TCGA-AC-A2FE-01A	C2
TCGA-AC-A2FK-01A	C2
TCGA-AR-A250-01A	C2
TCGA-BH-A18K-01A	C2
TCGA-BH-A1EO-01A	C2
TCGA-AR-A256-01A	C2
TCGA-AR-A1AP-01A	C2
TCGA-AO-A03P-01A	C2
TCGA-AR-A1AN-01A	C2
TCGA-AR-A24S-01A	C2
TCGA-A2-A0ST-01A	C2

TCGA-AR-A1AQ-01A	C2
TCGA-AC-A2BM-01A	C2
TCGA-GM-A2DN-01A	C2
TCGA-A2-A0EM-01A	C2
TCGA-AR-A24U-01A	C2
TCGA-AR-A24Q-01A	C2
TCGA-AR-A24T-01A	C2
TCGA-AO-A128-01A	C2
TCGA-A2-A25A-01A	C2
TCGA-A2-A0CW-01A	C2
TCGA-Z7-A8R5-01A	C2
TCGA-AR-A1AI-01A	C2
TCGA-AO-A126-01A	C2
TCGA-GI-A2C9-01A	C2
TCGA-B6-A0WY-01A	C2
TCGA-A2-A0EP-01A	C2
TCGA-AR-A24M-01A	C2
TCGA-BH-A1FB-01A	C2
TCGA-BH-A209-01A	C2
TCGA-3C-AALI-01A	C2
TCGA-AR-A0U3-01A	C2
TCGA-A2-A0EN-01A	C2
TCGA-B6-A0IQ-01A	C2
TCGA-B6-A0IO-01A	C2
TCGA-B6-A0WZ-01A	C2
TCGA-B6-A0RI-01A	C2
TCGA-A8-A06Q-01A	C3
TCGA-OK-A5Q2-01A	C3
TCGA-BH-A0HO-01A	C3
TCGA-E9-A1R3-01A	C3
TCGA-BH-A0DS-01A	C3
TCGA-LL-A6FQ-01A	C3
TCGA-BH-A18G-01A	C3
TCGA-A2-A0CU-01A	C3
TCGA-BH-A6R9-01A	C3
TCGA-AN-A0XV-01A	C3
TCGA-E9-A1R4-01A	C3
TCGA-AN-A0FD-01A	C3
TCGA-E9-A1RF-01A	C3
TCGA-BH-A18T-01A	C3
TCGA-EW-A1P8-01A	C3
TCGA-AC-A7VB-01A	C3
TCGA-E2-A56Z-01A	C3

TCGA-A1-A0SB-01A	C3
TCGA-A8-A09R-01A	C3
TCGA-E2-A15K-01A	C3
TCGA-BH-AB28-01A	C3
TCGA-BH-A6R8-01A	C3
TCGA-PL-A8LZ-01A	C3
TCGA-5T-A9QA-01A	C3
TCGA-LD-A9QF-01A	C3
TCGA-A8-A07P-01A	C3
TCGA-C8-A1HI-01A	C3
TCGA-A7-A3IY-01A	C3
TCGA-JL-A3YX-01A	C3
TCGA-BH-A1EV-01A	C3
TCGA-A8-A0A1-01A	C3
TCGA-A8-A08I-01A	C3
TCGA-A8-A07J-01A	C3
TCGA-C8-A1HK-01A	C3
TCGA-D8-A27W-01A	C3
TCGA-AC-A6IX-01A	C3
TCGA-C8-A1HE-01A	C3
TCGA-E9-A295-01A	C3
TCGA-C8-A137-01A	C3
TCGA-C8-A26W-01A	C3
TCGA-C8-A12Z-01A	C3
TCGA-C8-A132-01A	C3
TCGA-C8-A12W-01A	C3
TCGA-D8-A73W-01A	C3
TCGA-C8-A12X-01A	C3
TCGA-C8-A12Q-01A	C3
TCGA-C8-A12U-01A	C3
TCGA-C8-A3M8-01A	C3
TCGA-LL-A5YM-01A	C3
TCGA-C8-A8HP-01A	C3
TCGA-A8-A06P-01A	C3
TCGA-A8-A06O-01A	C3
TCGA-D8-A140-01A	C3
TCGA-LD-A7W6-01A	C3
TCGA-D8-A27M-01A	C3
TCGA-A1-A0SJ-01A	C3
TCGA-A8-A07I-01A	C3
TCGA-D8-A1Y2-01A	C3
TCGA-A7-A4SC-01A	C3
TCGA-A7-A56D-01A	C3

TCGA-LL-A5YP-01A	C3
TCGA-A7-A4SA-01A	C3
TCGA-AC-A5XU-01A	C3
TCGA-AC-A3TN-01A	C3
TCGA-D8-A1XV-01A	C3
TCGA-D8-A1XZ-01A	C3
TCGA-D8-A1Y0-01A	C3
TCGA-AQ-A1H2-01A	C3
TCGA-BH-A0DP-01A	C3
TCGA-D8-A27F-01A	C3
TCGA-A2-A1G6-01A	C3
TCGA-E2-A1BC-01A	C3
TCGA-D8-A1XT-01A	C3
TCGA-C8-A274-01A	C3
TCGA-C8-A273-01A	C3
TCGA-A8-A075-01A	C3
TCGA-E2-A15D-01A	C3
TCGA-OL-A66O-01A	C3
TCGA-AC-A62Y-01A	C3
TCGA-A2-A0T0-01A	C3
TCGA-D8-A1XM-01A	C3
TCGA-BH-A1EY-01A	C3
TCGA-D8-A1X6-01A	C3
TCGA-AO-A1KT-01A	C3
TCGA-A8-A093-01A	C3
TCGA-A2-A0YE-01A	C3
TCGA-EW-A1IZ-01A	C3
TCGA-E2-A14Z-01A	C3
TCGA-D8-A1X5-01A	C3
TCGA-S3-AA12-01A	C3
TCGA-A2-A0T6-01A	C3
TCGA-E9-A1NH-01A	C3
TCGA-A8-A0A2-01A	C3
TCGA-AC-A23C-01A	C3
TCGA-EW-A1PH-01A	C3
TCGA-E9-A243-01A	C3
TCGA-C8-A26V-01A	C3
TCGA-BH-A0AW-01A	C3
TCGA-A8-A0A6-01A	C3
TCGA-E2-A155-01A	C3
TCGA-AO-A0J3-01A	C3
TCGA-AO-A0JA-01A	C3
TCGA-E2-A1IN-01A	C3

TCGA-AC-A3QP-01A	C3
TCGA-MS-A51U-01A	C3
TCGA-EW-A1P5-01A	C3
TCGA-AC-A3YI-01A	C3
TCGA-AR-A0TU-01A	C3
TCGA-E2-A15A-01A	C3
TCGA-EW-A424-01A	C3
TCGA-E9-A54X-01A	C3
TCGA-BH-A0HF-01A	C3
TCGA-BH-A0W3-01A	C3
TCGA-AC-A3QQ-01B	C3
TCGA-A7-A26F-01B	C3
TCGA-E2-A1LA-01A	C3
TCGA-A2-A0CM-01A	C3
TCGA-AC-A3TM-01A	C3
TCGA-E9-A5UO-01A	C3
TCGA-AO-A0JG-01A	C3
TCGA-BH-A0HB-01A	C3
TCGA-BH-A18L-01A	C3
TCGA-E2-A2P5-01A	C3
TCGA-A8-A0A9-01A	C3
TCGA-A2-A0SV-01A	C3
TCGA-E2-A108-01A	C3
TCGA-A1-A0SO-01A	C3
TCGA-E9-A1R0-01A	C3
TCGA-E2-A10E-01A	C3
TCGA-E2-A1B6-01A	C3
TCGA-WT-AB44-01A	C3
TCGA-E9-A2JS-01A	C3
TCGA-E9-A3QA-01A	C3
TCGA-BH-A0EE-01A	C3
TCGA-EW-A6SC-01A	C3
TCGA-A7-A26E-01B	C3
TCGA-A2-A0YM-01A	C3
TCGA-E2-A14X-01A	C3
TCGA-E2-A14W-01A	C3
TCGA-E9-A227-01A	C3
TCGA-A8-A07L-01A	C3
TCGA-E2-A1B5-01A	C3
TCGA-BH-A0EA-01A	C3
TCGA-A8-A08S-01A	C3
TCGA-A8-A09M-01A	C3
TCGA-A7-A0DB-01A	C3

TCGA-BH-A1FD-01A	C3
TCGA-AR-A5QN-01A	C3
TCGA-D8-A3Z5-01A	C3
TCGA-E2-A1IH-01A	C3
TCGA-A2-A3XS-01A	C3
TCGA-C8-A3M7-01A	C3
TCGA-E2-A14V-01A	C3
TCGA-A2-A0D1-01A	C3
TCGA-OL-A5RV-01A	C3
TCGA-E2-A573-01A	C3
TCGA-E9-A1R2-01A	C3
TCGA-A7-A0DA-01A	C3
TCGA-OL-A5D6-01A	C3
TCGA-BH-A28Q-01A	C3
TCGA-BH-A0BA-01A	C3
TCGA-BH-A18R-01A	C3
TCGA-BH-A0DH-01A	C3
TCGA-AR-A2LN-01A	C3
TCGA-E2-A14R-01A	C3
TCGA-BH-A203-01A	C3
TCGA-BH-A0B4-01A	C3
TCGA-AR-A2LO-01A	C3
TCGA-E9-A1RC-01A	C3
TCGA-E2-A10A-01A	C3
TCGA-E9-A22H-01A	C3
TCGA-AC-A2FB-01A	C3
TCGA-E9-A22G-01A	C3
TCGA-E2-A14P-01A	C3
TCGA-E9-A22D-01A	C3
TCGA-BH-A0C0-01A	C3
TCGA-A2-A25B-01A	C3
TCGA-E2-A14U-01A	C3
TCGA-BH-A0C1-01B	C3
TCGA-E2-A109-01A	C3
TCGA-E2-A14N-01A	C3
TCGA-D8-A27K-01A	C3
TCGA-E9-A1R7-01A	C3
TCGA-C8-A12Y-01A	C3
TCGA-A8-A09E-01A	C3
TCGA-A2-A0T3-01A	C3
TCGA-BH-A0BV-01A	C3
TCGA-E2-A1LG-01A	C3
TCGA-A2-A0SX-01A	C3

TCGA-BH-A0HW-01A	C3
TCGA-BH-A18U-01A	C3
TCGA-BH-A0B8-01A	C3
TCGA-AO-A0J4-01A	C3
TCGA-EW-A2FS-01A	C3
TCGA-EW-A1P3-01A	C3
TCGA-BH-A0HA-01A	C3
TCGA-E2-A1B0-01A	C3
TCGA-AR-A2LK-01A	C3
TCGA-A2-A0SU-01A	C3
TCGA-BH-A1FL-01A	C3
TCGA-AR-A0TY-01A	C3
TCGA-D8-A13Y-01A	C3
TCGA-A2-A0CX-01A	C3
TCGA-AO-A03U-01B	C3
TCGA-AO-A12F-01A	C3
TCGA-AC-A2FG-01A	C3
TCGA-A2-A0D3-01A	C3
TCGA-BH-A0BM-01A	C3
TCGA-BH-A0AU-01A	C3
TCGA-A2-A04V-01A	C3
TCGA-BH-A1FJ-01A	C3
TCGA-AR-A2LM-01A	C3
TCGA-PE-A5DD-01A	C3
TCGA-AR-A0TX-01A	C3
TCGA-AO-A0JF-01A	C3
TCGA-AO-A03N-01B	C3
TCGA-BH-A0DG-01A	C3
TCGA-BH-A0DV-01A	C3
TCGA-E2-A14Y-01A	C3
TCGA-BH-A1EN-01A	C3
TCGA-E2-A1IG-01A	C3
TCGA-AR-A255-01A	C3
TCGA-BH-A18M-01A	C3
TCGA-B6-A1KI-01A	C3
TCGA-AC-A23G-01A	C3
TCGA-BH-A1FE-01A	C3
TCGA-E2-A14T-01A	C3
TCGA-E2-A1AZ-01A	C3
TCGA-BH-A0BR-01A	C3
TCGA-AO-A0JE-01A	C3
TCGA-A2-A04Q-01A	C3
TCGA-GM-A2DB-01A	C3

TCGA-BH-A0B0-01A	C3
TCGA-BH-A0B6-01A	C3
TCGA-BH-A0E9-01B	C3
TCGA-GM-A2DC-01A	C3
TCGA-AR-A1AO-01A	C3
TCGA-AR-A1AX-01A	C3
TCGA-GM-A2DK-01A	C3
TCGA-E2-A1B1-01A	C3
TCGA-BH-A1F5-01A	C3
TCGA-B6-A0RT-01A	C3
TCGA-AC-A2FF-01A	C3
TCGA-AR-A252-01A	C3
TCGA-AR-A1AU-01A	C3
TCGA-AO-A12B-01A	C3
TCGA-AR-A251-01A	C3
TCGA-AR-A24N-01A	C3
TCGA-B6-A0RS-01A	C3
TCGA-B6-A1KF-01A	C3
TCGA-A2-A04W-01A	C3
TCGA-B6-A40B-01A	C3
TCGA-AR-A1AK-01A	C3
TCGA-A2-A25E-01A	C3
TCGA-Z7-A8R6-01A	C3
TCGA-AR-A0U4-01A	C3
TCGA-A8-A08T-01A	C3
TCGA-AR-A24R-01A	C3
TCGA-BH-A1ES-01A	C3
TCGA-AR-A24O-01A	C3
TCGA-A2-A04R-01A	C3
TCGA-AR-A1AH-01A	C3
TCGA-AQ-A04L-01B	C3
TCGA-AR-A0TP-01A	C3
TCGA-B6-A0I2-01A	C3
TCGA-AR-A24H-01A	C3
TCGA-B6-A0RO-01A	C3
TCGA-B6-A2IU-01A	C3
TCGA-B6-A0WT-01A	C3
TCGA-B6-A0RH-01A	C3
TCGA-B6-A0IJ-01A	C3
TCGA-B6-A0I5-01A	C3
TCGA-A8-A06Z-01A	C4
TCGA-AN-A04D-01A	C4
TCGA-AC-A8OS-01A	C4

TCGA-BH-A0HL-01A	C4
TCGA-AR-A24P-01A	C4
TCGA-E9-A1R5-01A	C4
TCGA-BH-A0DQ-01A	C4
TCGA-UU-A93S-01A	C4
TCGA-AQ-A0Y5-01A	C4
TCGA-AN-A0FF-01A	C4
TCGA-AN-A0FN-01A	C4
TCGA-A2-A4RW-01A	C4
TCGA-A1-A0SM-01A	C4
TCGA-EW-A1IY-01A	C4
TCGA-A7-A6VY-01A	C4
TCGA-OL-A97C-01A	C4
TCGA-A8-A079-01A	C4
TCGA-A7-A6VW-01A	C4
TCGA-E9-A2JT-01A	C4
TCGA-BH-A8FY-01A	C4
TCGA-E9-A1NI-01A	C4
TCGA-D8-A1Y1-01A	C4
TCGA-C8-A1HF-01A	C4
TCGA-E2-A1IU-01A	C4
TCGA-AC-A62V-01A	C4
TCGA-A7-A426-01A	C4
TCGA-A8-A097-01A	C4
TCGA-D8-A1JF-01A	C4
TCGA-C8-A1HO-01A	C4
TCGA-AN-A0XO-01A	C4
TCGA-C8-A134-01A	C4
TCGA-D8-A1XU-01A	C4
TCGA-A8-A0A4-01A	C4
TCGA-D8-A27T-01A	C4
TCGA-GM-A5PV-01A	C4
TCGA-S3-AA17-01A	C4
TCGA-A1-A0SD-01A	C4
TCGA-D8-A27I-01A	C4
TCGA-C8-A27B-01A	C4
TCGA-LL-A5YO-01A	C4
TCGA-LL-A5YN-01A	C4
TCGA-D8-A1XG-01A	C4
TCGA-D8-A1XF-01A	C4
TCGA-LL-A6FR-01A	C4
TCGA-D8-A73U-01A	C4
TCGA-D8-A1X7-01A	C4

TCGA-AC-A5EH-01A	C4
TCGA-UL-AAZ6-01A	C4
TCGA-D8-A27N-01A	C4
TCGA-A2-A25C-01A	C4
TCGA-AR-A1AR-01A	C4
TCGA-S3-AA15-01A	C4
TCGA-LL-A9Q3-01A	C4
TCGA-GM-A5PX-01A	C4
TCGA-BH-A0BD-01A	C4
TCGA-BH-A5IZ-01A	C4
TCGA-A8-A07G-01A	C4
TCGA-AQ-A7U7-01A	C4
TCGA-AC-A2QI-01A	C4
TCGA-LL-A7SZ-01A	C4
TCGA-E2-A15P-01A	C4
TCGA-E2-A1L9-01A	C4
TCGA-A7-A13E-01A	C4
TCGA-AO-A0J7-01A	C4
TCGA-BH-A0HI-01A	C4
TCGA-S3-AA0Z-01A	C4
TCGA-A7-A26I-01B	C4
TCGA-D8-A1XJ-01A	C4
TCGA-E9-A1N6-01A	C4
TCGA-OL-A5RZ-01A	C4
TCGA-E2-A15C-01A	C4
TCGA-AC-A23E-01A	C4
TCGA-E2-A156-01A	C4
TCGA-D8-A1X9-01A	C4
TCGA-E9-A24A-01A	C4
TCGA-A2-A0EX-01A	C4
TCGA-OL-A5RY-01A	C4
TCGA-LL-A440-01A	C4
TCGA-A8-A09Q-01A	C4
TCGA-A2-A0YD-01A	C4
TCGA-BH-A0AY-01A	C4
TCGA-OL-A6VO-01A	C4
TCGA-OL-A5RX-01A	C4
TCGA-A8-A08C-01A	C4
TCGA-AC-A4ZE-01A	C4
TCGA-EW-A1OY-01A	C4
TCGA-EW-A1P7-01A	C4
TCGA-BH-A0GY-01A	C4
TCGA-A7-A0CJ-01A	C4

TCGA-A8-A08O-01A	C4
TCGA-OL-A5D8-01A	C4
TCGA-E9-A3Q9-01A	C4
TCGA-E2-A1II-01A	C4
TCGA-AR-A1AY-01A	C4
TCGA-A2-A0D2-01A	C4
TCGA-BH-A1FH-01A	C4
TCGA-E2-A2P6-01A	C4
TCGA-A2-A0ET-01A	C4
TCGA-E9-A1NE-01A	C4
TCGA-OL-A5RW-01A	C4
TCGA-BH-A0HQ-01A	C4
TCGA-A7-A0D9-01A	C4
TCGA-A8-A08B-01A	C4
TCGA-A8-A0AD-01A	C4
TCGA-E9-A247-01A	C4
TCGA-E9-A1NC-01A	C4
TCGA-E2-A572-01A	C4
TCGA-OL-A5RU-01A	C4
TCGA-OL-A6VR-01A	C4
TCGA-AR-A2LQ-01A	C4
TCGA-E9-A22E-01A	C4
TCGA-BH-A1EU-01A	C4
TCGA-A8-A07B-01A	C4
TCGA-A8-A08X-01A	C4
TCGA-E2-A105-01A	C4
TCGA-D8-A1XW-01A	C4
TCGA-E2-A3DX-01A	C4
TCGA-B6-A1KC-01B	C4
TCGA-A8-A09I-01A	C4
TCGA-A2-A3XX-01A	C4
TCGA-E9-A1RI-01A	C4
TCGA-A2-A0YI-01A	C4
TCGA-A8-A09D-01A	C4
TCGA-A2-A3Y0-01A	C4
TCGA-AR-A24K-01A	C4
TCGA-AR-A24W-01A	C4
TCGA-E2-A15T-01A	C4
TCGA-BH-A0B9-01A	C4
TCGA-A8-A06T-01A	C4
TCGA-AO-A12G-01A	C4
TCGA-A8-A076-01A	C4
TCGA-D8-A1JB-01A	C4

TCGA-BH-A18Q-01A	C4
TCGA-BH-A1EW-01A	C4
TCGA-E2-A15R-01A	C4
TCGA-BH-A0AV-01A	C4
TCGA-A2-A0EW-01A	C4
TCGA-B6-A0IE-01A	C4
TCGA-AO-A03T-01A	C4
TCGA-E2-A152-01A	C4
TCGA-AO-A12E-01A	C4
TCGA-GM-A2DF-01A	C4
TCGA-AC-A2FO-01A	C4
TCGA-BH-A0BL-01A	C4
TCGA-AR-A0TV-01A	C4
TCGA-E2-A1LB-01A	C4
TCGA-BH-A0DL-01A	C4
TCGA-BH-A0DT-01A	C4
TCGA-A2-A0EQ-01A	C4
TCGA-BH-A0B7-01A	C4
TCGA-GM-A2DI-01A	C4
TCGA-AR-A1AW-01A	C4
TCGA-AR-A2LJ-01A	C4
TCGA-PE-A5DE-01A	C4
TCGA-AR-A24L-01A	C4
TCGA-AO-A1KP-01A	C4
TCGA-AR-A1AL-01A	C4
TCGA-AR-A0TQ-01A	C4
TCGA-A2-A0CV-01A	C4
TCGA-AR-A24V-01A	C4
TCGA-A2-A0CR-01A	C4
TCGA-GM-A2DL-01A	C4
TCGA-B6-A0IP-01A	C4
TCGA-B6-A0IB-01A	C4
TCGA-A2-A0CK-01A	C4
TCGA-B6-A0RV-01A	C4
TCGA-B6-A0IA-01A	C4
TCGA-B6-A0RU-01A	C4

Supplementary Material S5: DMFS (Distant metastasis free survival) curve of AGO2, EIF4E3, and EIF4E

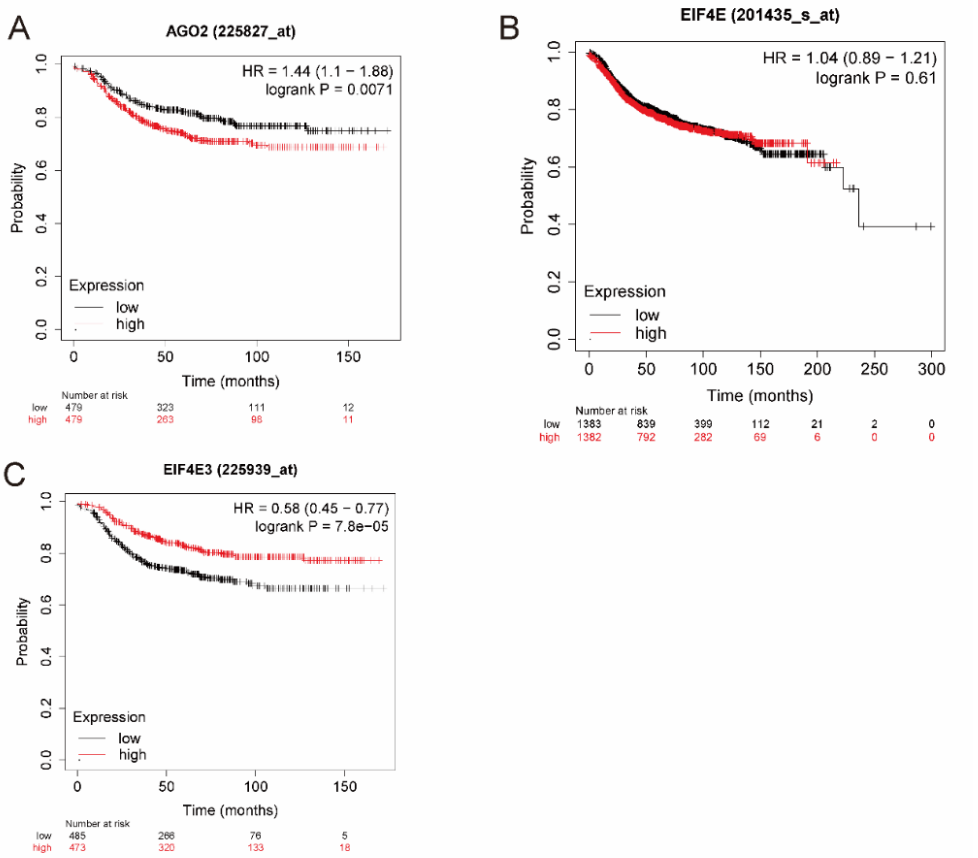

Figure 2 KM curve (DMFS) of AGO2 (A), EIF4E3 (B), and EIF4E (C).

Supplementary Material S6: C index of nomogram

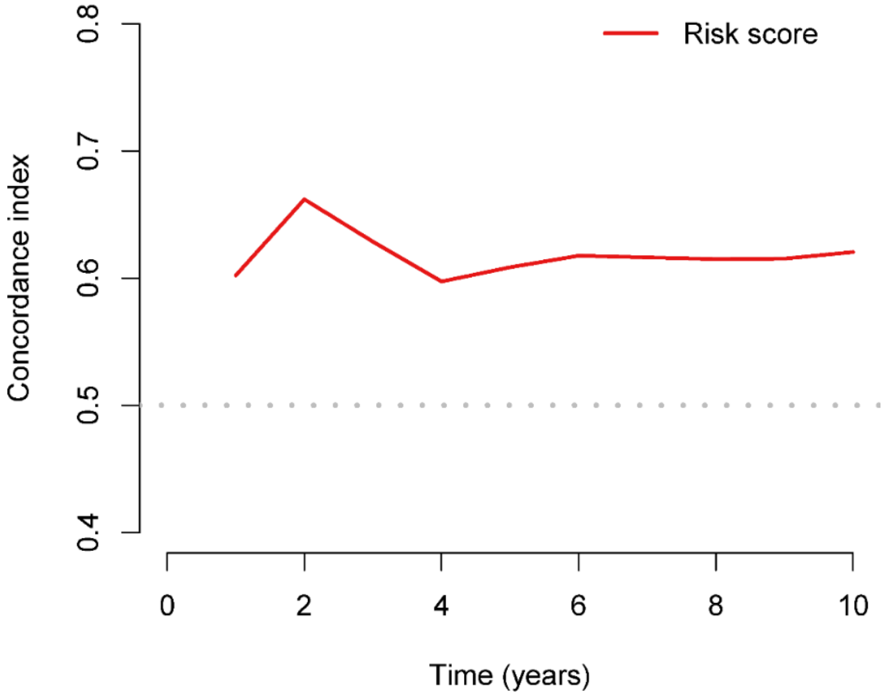

Figure 3 C index of nomogram

Supplementary Material S7: The expression landscapes of AGO2, EIF4E3, DCPS, and EIF4E in different cell lines based on the CCLE database.

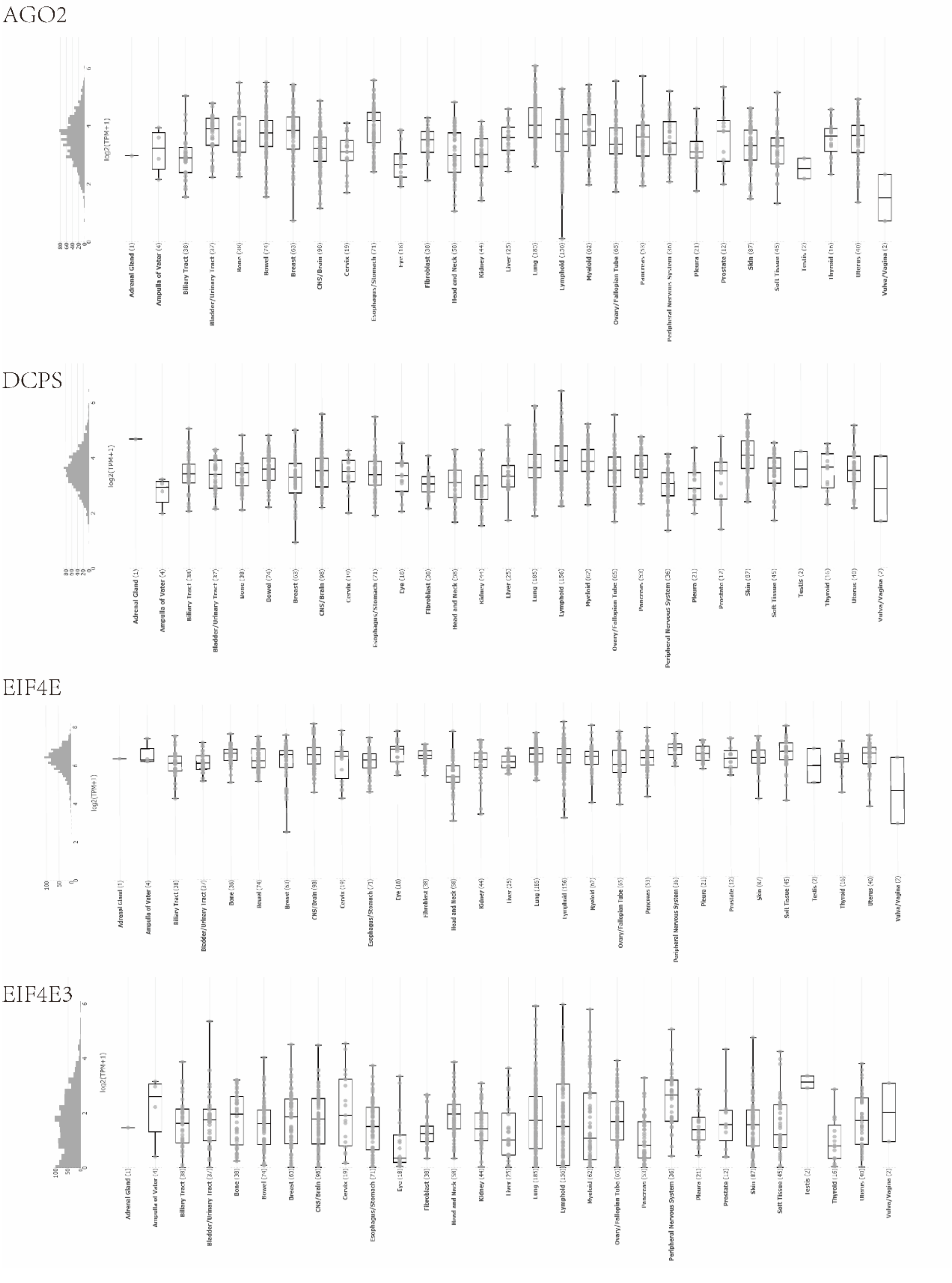

Figure 4 The expression landscapes of AGO2, EIF4E3, DCPS, and EIF4E in different cell lines based on the CCLE database.
